# Updated Trends in Cancer in Japan: Incidence in 1985–2015 and Mortality in 1958–2018—A Sign of Decrease in Cancer Incidence

**DOI:** 10.2188/jea.JE20200416

**Published:** 2021-07-05

**Authors:** Kota Katanoda, Megumi Hori, Eiko Saito, Akiko Shibata, Yuri Ito, Tetsuji Minami, Sayaka Ikeda, Tatsuya Suzuki, Tomohiro Matsuda

**Affiliations:** 1 Division of Cancer Statistics Integration, Center for Cancer Control and Information Services, National Cancer Center, Tokyo, Japan; 2 Center for Cancer Registries, Center for Cancer Control and Information Services, National Cancer Center, Tokyo, Japan; 3 Department of Medical Statistics, Research & Development Center, Osaka Medical and Pharmaceutical University, Osaka, Japan; 4 Department of Cancer Epidemiology, Division of Social Medicine, Graduate School of Medicine, The University of Tokyo, Tokyo, Japan; 5 Division of Prevention, Center for Public Health Sciences, National Cancer Center, Tokyo, Japan; 6 Department of Society and Environmental Medicine, Osaka University, Osaka, Japan; 7 Department of Hematology, National Cancer Center Hospital, Tokyo, Japan

**Keywords:** incidence, mortality, neoplasms, population surveillance, vital statistics

## Abstract

**Background:**

Unlike many North American and European countries, Japan has observed a continuous increase in cancer incidence over the last few decades. We examined the most recent trends in population-based cancer incidence and mortality in Japan.

**Methods:**

National cancer mortality data between 1958 and 2018 were obtained from published vital statistics. Cancer incidence data between 1985 and 2015 were obtained from high-quality population-based cancer registries maintained by three prefectures (Yamagata, Fukui, and Nagasaki). Trends in age-standardized rates (ASR) were examined using Joinpoint regression analysis.

**Results:**

For males, all-cancer incidence increased between 1985 and 1996 (annual percent change [APC] +1.1%; 95% confidence interval [CI], 0.7–1.5%), increased again in 2000–2010 (+1.3%; 95% CI, 0.9–1.8%), and then decreased until 2015 (−1.4%; 95% CI, −2.5 to −0.3%). For females, all-cancer incidence increased until 2010 (+0.8%; 95% CI, 0.6–0.9% in 1985–2004 and +2.4%; 95% CI, 1.3–3.4% in 2004–2010), and stabilized thereafter until 2015. The post-2000 increase was mainly attributable to prostate in males and breast in females, which slowed or levelled during the first decade of the 2000s. After a sustained increase, all-cancer mortality for males decreased in 1996–2013 (−1.6%; 95% CI, −1.6 to −1.5%) and accelerated thereafter until 2018 (−2.5%; 95% CI, −2.9 to −2.0%). All-cancer mortality for females decreased intermittently throughout the observation period, with the most recent APC of −1.0% (95% CI, −1.1 to −0.9%) in 2003–2018. The recent decreases in mortality in both sexes, and in incidence in males, were mainly attributable to stomach, liver, and male lung cancers.

**Conclusion:**

The ASR of all-cancer incidence began decreasing significantly in males and levelled off in females in 2010.

## INTRODUCTION

Globally, the incidence of major cancers is entering a decreasing phase. For example, a significant decrease in age-standardize rate (ASR) has been observed for colorectal, male lung, female breast, and cervical cancer incidence in North American^1^^,^^2^ and European countries,^3^^,^^4^ as well as in Asian populations.^5^ These decreasing trends have been interpreted as having resulted from effective cancer control policies, including tobacco control and screening interventions.^6^^–^^10^ By contrast, Japan is reported to be experiencing a significant increase in ASR of cancer incidence for all major cancer sites except stomach and liver.^11^^–^^13^ These reports are relatively outdated, however, with the most recent year of diagnosis being 2012, and subsequent trends in incidence in Japan have yet to be identified. Further, trends in cancers other than major cancer sites have not been sufficiently documented.^11^^–^^16^ Japan enacted the Cancer Registration Promotion Act in 2013, under which the registration of diagnosed cancer cases was started in 2016 under the new National Cancer Registry (NCR) system. Incidence data collected under the NCR to date has been published for diagnosed cases in 2016 and 2017 only, and at least a decade will be required before the secular trends in these national data can be evaluated. Accordingly, the present study aimed to examine secular trends in cancer incidence and mortality in Japan, including non-major cancer sites, with the use of the most recent data available from selected high-quality population-based cancer registries and national vital statistics.

## METHODS

Cancer incidence data were obtained in the framework of the Monitoring of Cancer Incidence in Japan (MCIJ) project.^17^^,^^18^ Annual cancer incidence data between 1985 and 2015 (before introduction of the NCR) were obtained from population-based cancer registries in four prefectures (Miyagi, Yamagata, Fukui, and Nagasaki), which were selected because of the availability of long-term high-quality data.^19^ Although we had confirmed the validity of using data from these four prefectural cancer registries in terms of representativeness for Japan,^19^ the updating of data from Miyagi Prefecture was unstable as a result of the ongoing data transfer process related to the start of NCR. Therefore, as adopted in our previous study,^11^^,^^12^ the present study used cancer incidence data from three prefectures (Yamagata, Fukui, and Nagasaki). The validity of using these three prefectures was previously confirmed.^12^

For cancer mortality, we obtained the population and number of annual cancer deaths between 1958 and 2018 from published vital statistics.^20^ Prefectural population data were obtained from the Center for Cancer Control and Information Services, National Cancer Center, Japan for the years 1985–2006,^21^ and from the Bureau of Statistics, Ministry of Internal Affairs and Communications for the years 2007–2015.^22^

We analyzed site-specific cancers and all cancers combined with reference to the International Classification of Diseases (ICD) version 10 codes (C00–C96, additionally D00–D09 for incidence, and C00–C97 for mortality). Twenty-five cancer sites were selected according to the list of cancers adopted by the National Cancer Center, Japan,^23^ which were the same as those analyzed in our previous analysis.^11^^,^^12^ We defined “major cancer sites” as the five leading cancers, and “sub-major cancer sites” as the sixth to tenth leading cancers in the latest cancer statistics (either in males or females and in incidence or mortality).^23^ Specifically, major cancer sites were stomach, colon/rectum, liver, pancreas, lung, female breast, uterus, and prostate; sub-major cancer cites were esophagus, gallbladder and bile ducts, ovary, urinary bladder, kidney and other urinary organs (except bladder), thyroid, and malignant lymphoma. For these major and sub-major cancers, we discussed potential factors underlying the observed trends in incidence and mortality. We added the analysis of all-cancer incidence excluding stomach, stomach and liver, prostate, and female breast to examine the effect of these influential cancer sites. ASR were standardized to the 1985 model Japanese population for cancer incidence and mortality.

A Joinpoint regression model^24^ was applied using the Joinpoint Regression Program version 4.7.0.0, developed by the United States National Cancer Institute. In the Joinpoint Regression analysis, the number of incidence or death was assumed to follow a Poisson distribution; the maximum number of joinpoints was set at five; the minimum number of observations from a joinpoint to either end of the data was set at two; and the minimum number of observations between two joinpoints was set at three.

To identify cancer sites contributing to the recent decrease in all-cancer mortality rates, we calculated the degree of contribution of each cancer site using the same method as that adopted in our previous study.^12^ Briefly, we calculated the average APC (AAPC) during the last 10 years for the trend of all-cancer and site-specific ASR of mortality for each sex, calculated the amount of change in ASR by multiplying the 10th power of (1+AAPC) by the ASR in the first year of the 10-year period, and then calculated the proportion of each cancer site in terms of the amount of change. For cancer incidence, since a joinpoint was observed during the last 10 years for both sexes (more specifically, the trend changed from significant increase to significant decrease or levelling off), the contribution of each cancer site was calculated using the same method as that used for cancer mortality, during each of the last significant increasing segment and the subsequent decreasing segment (if significant).

This study was approved by the institutional review board of the National Cancer Center, Japan (2019-202).

## RESULTS

Figure 1A shows annual trends in ASRs of incidence for all-cancer and major cancer sites in the three selected prefectures in Japan. The ASR of all-cancer incidence for males was 376.2 in 1985, which then peaked at 464.5 in 2010 and decreased to 431.8 in 2015. For females, the ASR was 228.3 in 1985, which then peaked at 307.5 in 2013 and decreased to 302.2 in 2015. Trends for sub-major cancers are shown in Figure 1B.

**Figure 1A.**
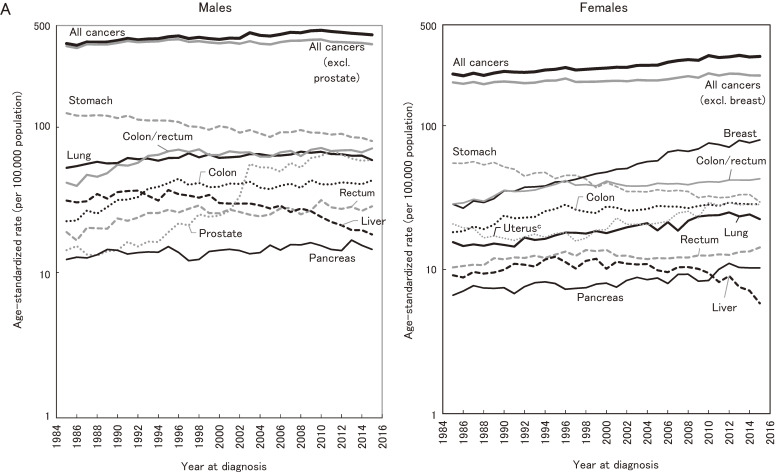
Annual trends in age-standardized rates of all-cancer and site-specific cancer incidence: data from three prefectures (1985–2015): Major sites^a,b^. ^a^Yamagata, Fukui, and Nagasaki Prefectures. ^b^Standardized to the Japanese model population in 1985. ^c^Cervix and corpus uteri are shown in Figure 1B.

**Figure 1B.**
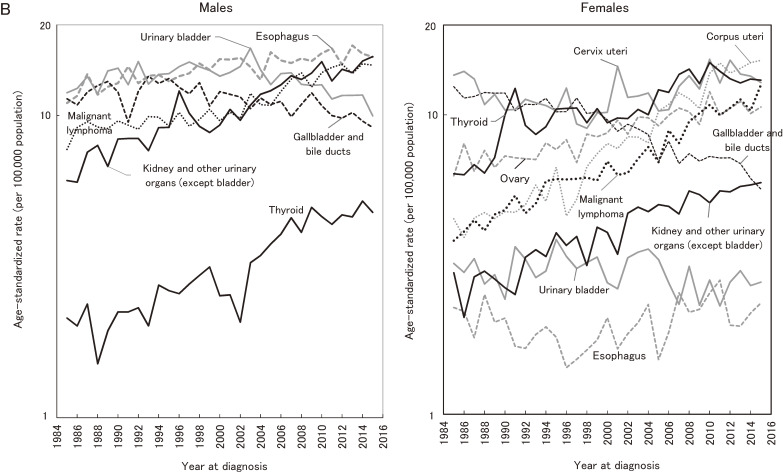
Annual trends in the age-standardized rates of all-cancer and site-specific cancer incidence: data from three prefectures (1985–2015): Sub-major sites^a,b^. ^a^Yamagata, Fukui, and Nagasaki Prefectures. ^b^Standardized to the Japanese model population in 1985.

Table 1 shows the results of the Joinpoint regression analysis on the trends in all-cancer incidence in the three selected prefectures. For males and females combined, the ASR of all-cancer incidence intermittently increased from 1985 through 2010 with a significant APC of +1.0% between 1985–1996 and +1.7% between 2000–2010, and then levelled off after 2010. Similar patterns were seen in the separate analyses for males and females; for males the decrease after 2010 was statistically significant. Overall results were the same when stomach and liver cancers were excluded, when prostate or female breast cancer were excluded (Table 1) or when the data were restricted to ages under 75 years old (eTable 1). Corresponding results that included carcinoma in situ are shown in eTable 2.

**Table 1.** Results of joinpoint regression analysis on the trends in all-cancer incidence: data from three prefectures (1985–2015)^a^

Age	Sex	Cancer site	ICD-10	Number of joinpoints	Line segment		Annual % change	95% confidence interval		
	
					Start	End		Lower	Upper	
All ages	Male and female	All cancers	C00–C96	3	1985	1996	1.0	0.7	1.3	^*^
					1996	2000	−0.5	−2.6	1.6	
					2000	2010	1.7	1.3	2.0	^*^
					2010	2015	−0.5	−1.4	0.4	
		All cancers excluding stomach	C00–C96 (excluding C16)	3	1985	1996	1.9	1.6	2.3	^*^
					1996	2000	0.2	−2.1	2.6	
					2000	2010	2.2	1.8	2.6	^*^
					2010	2015	−0.3	−1.3	0.6	
		All cancers excluding stomach and liver	C00–C96 (excluding C16 and C22)	2	1985	2005	1.7	1.5	1.9	^*^
					2005	2010	2.8	1.1	4.6	^*^
					2010	2015	−0.1	−1.3	1.1	

	Male	All cancers	C00–C96	3	1985	1996	1.1	0.7	1.5	^*^
					1996	2000	−0.9	−3.6	1.9	
					2000	2010	1.3	0.9	1.8	^*^
					2010	2015	−1.4	−2.5	−0.3	^*^
		All cancers excluding stomach	C00–C96 (excluding C16)	3	1985	1996	2.1	1.6	2.6	^*^
					1996	2000	−0.2	−3.4	3.1	
					2000	2010	1.8	1.3	2.4	^*^
					2010	2015	−1.3	−2.6	0.0	^*^
		All cancers excluding stomach and liver	C00–C96 (excluding C16 and C22)	1	1985	2010	1.8	1.6	1.9	^*^
					2010	2015	−0.6	−2.1	0.9	
		All cancers excluding prostate	C00–C96 (excluding C61)	3	1985	1995	1.0	0.6	1.4	^*^
					1995	2005	−0.6	−1.0	−0.2	^*^
					2005	2009	1.8	−0.4	4.0	
					2009	2015	−1.3	−2.0	−0.5	^*^

	Female	All cancers	C00–C96	2	1985	2004	0.8	0.6	0.9	^*^
					2004	2010	2.4	1.3	3.4	^*^
					2010	2015	0.2	−0.8	1.2	
		All cancers excluding stomach	C00–C96 (excluding C16)	2	1985	2005	1.6	1.4	1.7	^*^
					2005	2010	3.0	1.4	4.7	^*^
					2010	2015	0.3	−0.8	1.4	
		All cancers excluding stomach and liver	C00–C96 (excluding C16 and C22)	2	1985	2005	1.6	1.5	1.8	^*^
					2005	2010	3.3	1.7	4.9	^*^
					2010	2015	0.5	−0.6	1.6	
		All cancers excluding breast	C00–C96 (excluding C50)	2	1985	2005	0.2	0.1	0.4	^*^
					2005	2012	1.6	0.7	2.4	^*^
					2012	2015	−1.2	−3.8	1.3	

ICD-10, International Classification of Diseases, 10th revision.

^a^Yamagata, Fukui, and Nagasaki Prefectures.

^*^Annual % change was statistically significantly different from zero (*P* < 0.05).

Table 2A and Table 2B show the corresponding site-specific results of cancer incidence for males and females, respectively. For major cancer sites in males, pancreatic and prostate cancers showed significant increases through the observation period. The increase in pancreatic cancer was monotonous. On the other hand, prostate cancer significantly increased in the most recent segment, but the APC was much smaller than in the previous segment (1.3% in 2004–2015 vs 22.4% in 2000–2004). Major cancers that showed a significant decrease during the most recent segment (the period including the most recent year) were stomach, liver, and lung. Among sub-major cancer sites, esophagus, kidney and other urinary organs except bladder, and malignant lymphoma showed a significant increase in the most recent segment, while gallbladder and bile ducts and urinary bladder showed a significant decrease. For females, significantly increasing major cancers in the most recent segment were colon, rectum, (also colon and rectum combined), pancreas, lung, cervix uteri, and corpus uteri (also uterus as a whole). Notably, a long-term increase in breast cancer (APC 4.0% in 1985–2010) stopped in 2010. Thyroid cancer significantly increased in 2002–2008 (APC 6.5%), but levelled off thereafter. Significant decreases were seen in the most recent segment for stomach and liver. Among sub-major cancer sites, esophagus, ovary, kidney, and malignant lymphoma showed significant increases in the most recent segment, while gallbladder and urinary bladder showed a significant decrease. Among the other cancer sites, oral cavity and pharynx, skin and multiple myeloma showed a significant increase in both sexes.

**Table 2A.** Results of joinpoint regression analysis on the trends in site-specific cancer incidence: data from three prefectures (1985–2015); Male^a^

Cancer site		ICD-10	Number of joinpoints	Line segment		Annual % change	95% confidence interval		
	
				Start	End		Lower	Upper	
Major									
	Stomach	C16	2	1985	2005	−1.7	−1.9	−1.5	^*^
				2005	2009	1.1	−2.6	4.8	
				2009	2015	−2.4	−3.6	−1.1	^*^
	Colon	C18	1	1985	1995	6.1	4.8	7.4	^*^
				1995	2015	0.0	−0.3	0.3	
	Rectum	C19–C20	1	1985	1994	4.3	2.3	6.2	^*^
				1994	2015	0.3	−0.1	0.7	
	Colon/rectum	C18–C20	1	1985	1994	5.8	4.4	7.2	^*^
				1994	2015	0.2	−0.1	0.5	
	Liver	C22	2	1985	1991	3.3	0.9	5.8	^*^
				1991	2008	−1.7	−2.2	−1.3	^*^
				2008	2015	−5.7	−7.5	−3.9	^*^
	Pancreas	C25	0	1985	2015	0.6	0.3	0.8	^*^
	Lung, trachea	C33–C34	1	1985	2010	0.8	0.6	0.9	^*^
				2010	2015	−2.1	−3.8	−0.5	^*^
	Prostate	C61	2	1985	2000	4.7	3.0	6.5	^*^
				2000	2004	22.4	8.5	38.1	^*^
				2004	2015	1.3	0.0	2.6	^*^
Sub-major									
	Esophagus	C15	0	1985	2015	1.0	0.7	1.2	^*^
	Gallbladder and bile ducts	C23–C24	0	1985	2015	−0.8	−1.1	−0.4	^*^
	Urinary bladder	C67	1	1985	2003	0.9	0.3	1.4	^*^
				2003	2015	−2.7	−3.6	−1.9	^*^
	Kidney and other urinary organs (except bladder)	C64–C66 C68	0	1985	2015	2.8	2.4	3.2	^*^
	Thyroid	C73	2	1985	2002	2.0	0.9	3.2	^*^
				2002	2007	9.5	1.1	18.5	^*^
				2007	2015	1.1	−1.4	3.8	
	Malignant lymphoma	C81–C85 C96	1	1985	2000	0.9	0.1	1.7	^*^
				2000	2015	2.9	2.2	3.6	^*^
Others									
	Oral cavity and pharynx	C00–C14	0	1985	2015	2.3	1.9	2.8	^*^
	Larynx	C32	0	1985	2015	−0.5	−1.0	0.0	^*^
	Skin	C43–C44	0	1985	2015	2.6	2.1	3.1	^*^
	Breast	C50	0	1985	2015	1.7	0.2	3.2	^*^
	Brain, nervous system	C70–C72	0	1985	2015	−0.1	−0.6	0.5	
	Multiple myeloma	C88–C90	0	1985	2015	0.8	0.4	1.2	^*^
	Leukemia	C91–C95	0	1985	2015	0.0	−0.3	0.3	

ICD-10, International Classification of Diseases, 10th revision.

^a^Yamagata, Fukui, and Nagasaki Prefectures.

^*^Annual % change was statistically significantly different from zero (*P* < 0.05).

**Table 2B.** Results of joinpoint regression analysis on the trends in site-specific cancer incidence: data from three prefectures (1985–2015); Female^a^

Cancer site		ICD-10	Number of joinpoints	Line segment		Annual % change	95% confidence interval		
	
				Start	End		Lower	Upper	
Major									
	Stomach	C16	1	1985	2003	−2.6	−3.0	−2.2	^*^
				2003	2015	−1.2	−2.0	−0.4	^*^
	Colon	C18	1	1985	1995	3.6	2.6	4.6	^*^
				1995	2015	0.5	0.2	0.8	^*^
	Rectum	C19–C20	2	1985	1999	1.9	1.5	2.3	^*^
				1999	2004	−3.2	−5.5	−0.7	^*^
				2004	2015	1.4	0.9	2.0	^*^
	Colon/rectum	C18–C20	2	1985	1996	3.2	2.6	3.7	^*^
				1996	2003	−0.7	−1.8	0.4	
				2003	2015	0.9	0.5	1.3	^*^
	Liver	C22	2	1985	1995	2.8	1.4	4.2	^*^
				1995	2010	−1.2	−1.9	−0.4	^*^
				2010	2015	−8.5	−12.2	−4.7	^*^
	Pancreas	C25	0	1985	2015	1.3	1.0	1.6	^*^
	Lung, trachea	C33–C34	0	1985	2015	1.9	1.6	2.1	^*^
	Breast	C50	1	1985	2010	4.0	3.8	4.2	^*^
				2010	2015	1.7	−0.2	3.6	
	Uterus	C53–C55	1	1985	1991	−4.6	−7.8	−1.4	^*^
				1991	2015	2.8	2.3	3.2	^*^
	Cervix uteri	C53	1	1985	1991	−5.3	−9.8	−0.6	^*^
				1991	2015	1.3	0.7	2.0	^*^
	Corpus uteri	C54	0	1985	2015	5.0	4.5	5.4	^*^
Sub-major									
	Esophagus	C15	1	1985	1996	−2.8	−5.3	−0.1	^*^
				1996	2015	2.1	0.9	3.2	^*^
	Gallbladder and bile ducts	C23–C24	1	1985	1997	−1.1	−2.0	−0.2	^*^
				1997	2015	−3.0	−3.5	−2.5	^*^
	Ovary	C56	0	1985	2015	1.8	1.4	2.1	^*^
	Urinary bladder	C67	0	1985	2015	−0.6	−1.1	0.0	^*^
	Kidney and other urinary organs (except bladder)	C64–C66 C68	0	1985	2015	2.9	2.5	3.3	^*^
	Thyroid	C73	3	1985	1991	10.3	5.5	15.4	^*^
				1991	2002	−0.5	−2.3	1.4	
				2002	2008	6.5	1.4	11.8	^*^
				2008	2015	−1.2	−3.9	1.7	
	Malignant lymphoma	C81–C85 C96	0	1985	2015	3.7	3.4	4.1	^*^
Others									
	Oral cavity and pharynx	C00–C14	0	1985	2015	2.2	1.5	2.8	^*^
	Larynx	C32	0	1985	2015	0.6	−1.3	2.7	
	Skin	C43–C44	2	1985	1997	−1.3	−2.6	0.0	
				1997	2003	6.9	2.1	11.8	^*^
				2003	2015	1.9	0.9	3.0	^*^
	Brain, nervous system	C70–C72	0	1985	2015	−0.6	−1.4	0.1	
	Multiple myeloma	C88–C90	0	1985	2015	0.8	0.4	1.2	^*^
	Leukemia	C91–C95	0	1985	2015	0.3	−0.2	0.8	

ICD-10, International Classification of Diseases, 10th revision.

^a^Yamagata, Fukui, and Nagasaki Prefectures.

^*^Annual % change was statistically significantly different from zero (*P* < 0.05).

**Figure 2.**
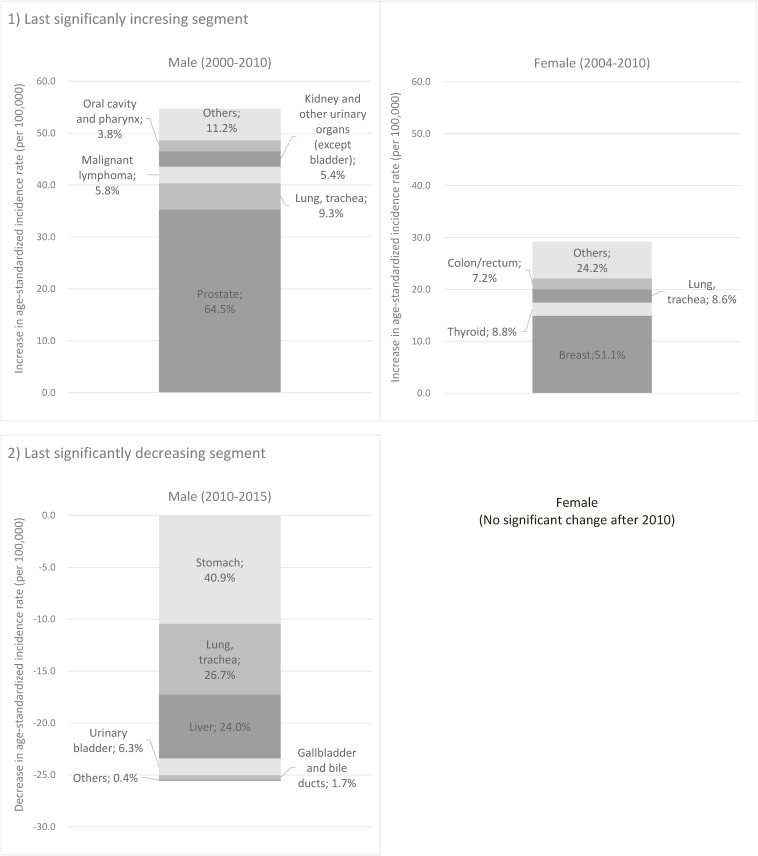
Contribution of cancer sites to changes in incidence.

Figure 2 shows the contribution of cancer sites to the significant increase or decrease in all-cancer incidence. For males, prostate cancer accounted for 64.5% of the most recent significant increase in all-cancer incidence between 2000 and 2010. The contributions of other cancer sites were less than 10% (lung: 9.3%, malignant lymphoma: 5.8%, kidney and other urinary organs except bladder: 5.4%, and oral cavity and pharynx: 3.8%). For females, the largest contribution of cancer site to the significant all-cancer increase in incidence between 2004 and 2010 was breast (51.1%), followed by thyroid (8.8%), lung (8.6%), and colon/rectum (7.2%). For males, all-cancer incidence significantly decreased after 2010, and this decrease was mainly accounted for by stomach (41.1%), lung (26.8%), and liver (24.1%) cancers. For females, there was no significant increase in all-cancer incidence after 2010.

Figure 3A shows the annual trends in ASRs of mortality for all-cancer and major cancer sites in Japan (national data). The ASR of all-cancer mortality for males was 182.6 in 1958, which then peaked at 226.1 in 1995 and decreased to 152.1 in 2018. For females, the ASR was 130.7 in 1958, which then peaked at 132.0 in 1960 and decreased to 84.5 in 2015. Trends of sub-major cancers are shown in Figure 3B.

**Figure 3A.**
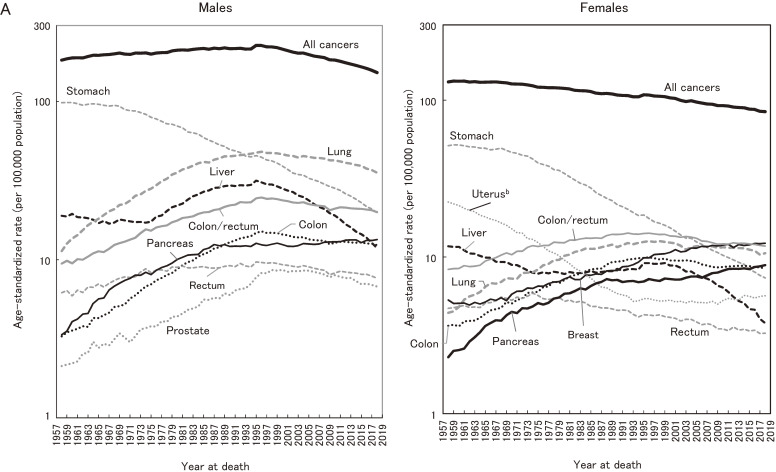
Annual trends in age-standardized rates of all-cancer and site-specific cancer mortality: national data (1958–2018): Major sites^a^. ^a^Standardized to the Japanese model population in 1985. ^b^Cervix and corpus uteri are shown in Figure 3B.

**Figure 3B.**
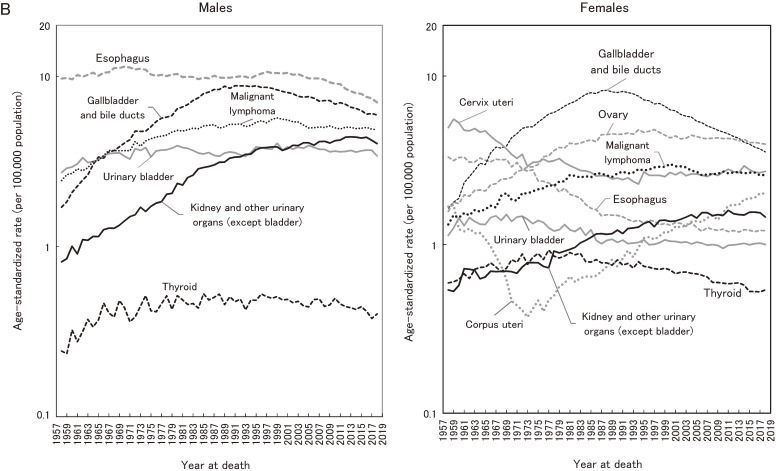
Annual trends in the age-standardized rates of all-cancer and site-specific cancer mortality: national data (1958–2018): Sub-major sites^a^. ^a^Standardized to the Japanese model population in 1985.

Table 3 shows the results of the Joinpoint regression analysis of all-cancer mortality from the national data of Japan. For males, the ASR of all-cancer mortality intermittently increased from 1958 through 1996 and decreased thereafter. The decrease accelerated from 2013 from the APC of −1.6% (1996–2013) to −2.5% (2013–2018). For females, the ASR of all-cancer mortality showed a long-term decreasing trend from 1968, with the APCs of −0.8% (1968–1993), −1.4% (1997–2003), and −1.0% (2003–2018). A similar decreasing trend was seen for males and females combined, with the APCs of −0.2% (1966–1993), −1.4% (1997–2015), and −2.2% (2015–2018). Overall results were the same when the data were restricted to ages under 75 years old (eTable 3).

**Table 3.** Results of joinpoint regression analysis on the trends in all-cancer mortality: national data (1958–2018)

Age	Sex	Cancer site	ICD-10	Number of joinpoints	Line segment		Annual % change	95% confidence interval		
	
					Start	End		Lower	Upper	
All ages	Male and female	All cancers	C00–C97	4	1958	1966	0.4	0.1	0.8	^*^
					1966	1993	−0.2	−0.2	−0.1	^*^
					1993	1997	0.6	−0.3	1.5	
					1997	2015	−1.4	−1.4	−1.3	^*^
					2015	2018	−2.2	−3.0	−1.4	^*^

		All cancers excluding stomach	C00–C97 (excluding C16)	5	1958	1975	1.1	1.0	1.2	^*^
					1975	1984	1.7	1.5	1.9	^*^
					1984	1993	0.7	0.5	0.9	^*^
					1993	1997	1.4	0.6	2.2	^*^
					1997	2016	−1.0	−1.1	−1.0	^*^
					2016	2018	−2.3	−3.8	−0.9	^*^

		All cancers excluding stomach and liver	C00–C97 (excluding C16 and C22)	4	1958	1984	1.5	1.4	1.5	^*^
					1984	1992	0.5	0.2	0.7	^*^
					1992	1996	1.6	0.7	2.5	^*^
					1996	2015	−0.6	−0.6	−0.5	^*^
					2015	2018	−1.4	−2.2	−0.6	^*^

	Male	All cancers	C00–C97	4	1958	1988	0.5	0.5	0.6	^*^
					1988	1992	−0.5	−1.7	0.7	
					1992	1996	1.5	0.4	2.7	^*^
					1996	2013	−1.6	−1.6	−1.5	^*^
					2013	2018	−2.5	−2.9	−2.0	^*^

		All cancers excluding stomach	C00–C97 (excluding C16)	4	1958	1975	2.0	1.8	2.2	^*^
					1975	1983	2.8	2.3	3.3	^*^
					1983	1997	1.3	1.2	1.4	^*^
					1997	2013	−1.3	−1.3	−1.2	^*^
					2013	2018	−2.1	−2.5	−1.6	^*^

		All cancers excluding stomach and liver	C00–C97 (excluding C16 and C22)	4	1958	1963	3.6	2.2	5.0	^*^
					1963	1982	2.4	2.3	2.5	^*^
					1982	1997	1.3	1.2	1.5	^*^
					1997	2014	−0.8	−0.9	−0.7	^*^
					2014	2018	−1.9	−2.5	−1.2	^*^

		All cancers excluding prostate	C00–C97 (excluding C61)	4	1958	1987	0.5	0.4	0.5	^*^
					1987	1992	−0.4	−1.1	0.4	
					1992	1996	1.2	0.1	2.4	^*^
					1996	2013	−1.6	−1.7	−1.5	^*^
					2013	2018	−2.5	−2.9	−2.0	^*^

	Female	All cancers	C00–C97	4	1958	1968	−0.1	−0.3	0.1	
					1968	1993	−0.8	−0.9	−0.8	^*^
					1993	1997	0.5	−0.4	1.4	
					1997	2003	−1.4	−1.8	−1.0	^*^
					2003	2018	−1.0	−1.1	−0.9	^*^

		All cancers excluding stomach	C00–C97 (excluding C16)	4	1958	1976	0.1	0.0	0.2	
					1976	1980	1.0	−0.2	2.2	
					1980	1993	0.2	0.0	0.3	^*^
					1993	1997	0.9	0.0	1.9	^*^
					1997	2018	−0.7	−0.8	−0.7	^*^

		All cancers excluding stomach and liver	C00–C97 (excluding C16 and C22)	5	1958	1976	0.4	0.3	0.5	^*^
					1976	1981	0.9	0.2	1.7	^*^
					1981	1993	0.1	−0.1	0.2	
					1993	1997	1.0	0.1	1.9	^*^
					1997	2003	−0.8	−1.2	−0.4	^*^
					2003	2018	−0.3	−0.4	−0.2	^*^

		All cancers excluding breast	C00–C97 (excluding C50)	5	1958	1968	−0.2	−0.4	0.0	
					1968	1993	−1.0	−1.0	−1.0	^*^
					1993	1997	0.1	−0.8	0.9	
					1997	2007	−1.6	−1.7	−1.4	^*^
					2007	2014	−1.0	−1.3	−0.7	^*^
					2014	2018	−1.7	−2.3	−1.1	^*^

ICD-10, International Classification of Diseases, 10th revision.

^*^Annual % change was statistically significantly different from zero (*P* < 0.05).

Table 4A and Table 4B show the corresponding site-specific results of cancer mortality for males and females, respectively. For major cancer sites in males, pancreatic cancer alone showed a significant increase during the most recent segment. Conversely, all the remaining major cancer sites showed a significant decrease during the most recent segment: stomach, colon, rectum (also colon and rectum combined), liver, lung, and prostate. Among sub-major cancer sites also, all cancer sites showed a significant decrease during the most recent segment, except malignant lymphoma, which significantly decreased in 2001–2005. For females, significantly increasing major cancers in the most recent segment were pancreas, breast, cervix uteri, and corpus uteri, (also uterus as a whole). Similarly to males, all the remaining major cancer sites showed a significant decrease during the most recent segment, except colon, which significantly decreased in 1993–2008 and levelled off thereafter. Among sub-major cancer sites, all cancer sites except kidney and malignant lymphoma showed a significant decrease during the most recent segment.

**Table 4A.** Results of joinpoint regression analysis on the trends in site-specific cancer mortality: national data (1958–2018); Male

Cancer site		ICD-10	Number of joinpoints	Line segment		Annual % change	95% confidence interval		
	
				Start	End		Lower	Upper	
Major									
	Stomach	C16	5	1958	1969	−0.5	−0.7	−0.3	^*^
				1969	1981	−2.6	−2.9	−2.4	^*^
				1981	1992	−3.3	−3.6	−3.1	^*^
				1992	1996	−1.4	−2.9	0.2	
				1996	2012	−3.2	−3.3	−3.1	^*^
				2012	2018	−4.6	−5.1	−4.0	^*^
	Colon	C18	4	1958	1985	4.7	4.6	4.9	^*^
				1985	1996	3.0	2.7	3.2	^*^
				1996	2009	−1.3	−1.4	−1.1	^*^
				2009	2015	0.1	−0.4	0.6	
				2015	2018	−1.3	−2.4	−0.2	^*^
	Rectum	C19–C20	2	1958	1975	2.2	1.8	2.5	^*^
				1975	1998	0.5	0.3	0.6	^*^
				1998	2018	−1.0	−1.1	−0.9	^*^
	Colon/rectum	C18–C20	3	1958	1980	3.1	2.9	3.3	^*^
				1980	1996	2.1	1.9	2.2	^*^
				1996	2009	−1.1	−1.3	−1.0	^*^
				2009	2018	−0.5	−0.7	−0.3	^*^
	Liver	C22	5	1958	1974	−0.6	−0.9	−0.3	^*^
				1974	1986	4.2	3.9	4.6	^*^
				1986	1996	1.0	0.6	1.3	^*^
				1996	2002	−2.3	−3.1	−1.6	^*^
				2002	2010	−4.2	−4.7	−3.8	^*^
				2010	2018	−5.5	−5.9	−5.1	^*^
	Pancreas	C25	3	1958	1968	7.3	6.4	8.2	^*^
				1968	1987	3.1	2.9	3.3	^*^
				1987	2002	0.1	−0.1	0.3	
				2002	2018	0.5	0.4	0.6	^*^
	Lung, trachea	C33–C34	5	1958	1963	7.5	5.8	9.2	^*^
				1963	1981	4.6	4.4	4.8	^*^
				1981	1989	2.2	1.9	2.6	^*^
				1989	1996	1.0	0.6	1.4	^*^
				1996	2012	−0.9	−1.0	−0.8	^*^
				2012	2018	−2.5	−2.8	−2.2	^*^
	Prostate	C61	3	1958	1993	3.3	3.1	3.4	^*^
				1993	1997	5.7	2.8	8.7	^*^
				1997	2005	0.1	−0.6	0.7	
				2005	2018	−1.7	−1.9	−1.5	^*^
Sub-major									
	Esophagus	C15	5	1958	1971	1.4	1.0	1.7	^*^
				1971	1977	−2.1	−3.3	−0.8	^*^
				1977	1994	−0.1	−0.3	0.1	
				1994	1998	2.0	0.1	4.0	^*^
				1998	2008	−1.1	−1.4	−0.7	^*^
				2008	2018	−2.9	−3.2	−2.6	^*^
	Urinary bladder	C67	3	1958	1980	1.2	0.9	1.5	^*^
				1980	1988	−1.6	−2.9	−0.3	^*^
				1988	1999	1.2	0.5	1.8	^*^
				1999	2018	−0.4	−0.6	−0.3	^*^
	Kidney and other urinary organs (except bladder)	C64–C66 C68	4	1958	1977	4.1	3.7	4.6	^*^
				1977	1984	6.0	4.5	7.6	^*^
				1984	1996	2.3	1.9	2.7	^*^
				1996	2016	0.9	0.7	1.0	^*^
				2016	2018	−4.7	−8.4	−0.8	^*^
	Thyroid	C73	2	1958	1966	7.4	3.3	11.7	^*^
				1966	1997	0.6	0.2	0.9	^*^
				1997	2018	−1.0	−1.3	−0.6	^*^
	Malignant lymphoma	C81–C85 C96	4	1958	1967	4.0	3.0	4.9	^*^
				1967	1980	2.4	2.0	2.9	^*^
				1980	2001	0.7	0.6	0.9	^*^
				2001	2005	−2.6	−4.8	−0.4	^*^
				2005	2018	−0.2	−0.4	0.0	
Others									
	Oral cavity and pharynx	C00–C14	3	1958	1992	1.8	1.7	2.0	^*^
				1992	1996	7.6	3.8	11.5	^*^
				1996	2008	0.5	0.1	0.9	^*^
				2008	2018	−0.7	−1.1	−0.3	^*^
	Gallbladder and bile ducts	C23–C24	4	1958	1965	10.1	8.3	12.0	^*^
				1965	1987	4.4	4.2	4.6	^*^
				1987	1995	0.4	−0.1	1.0	
				1995	2012	−1.5	−1.7	−1.4	^*^
				2012	2018	−2.3	−2.9	−1.8	^*^
	Larynx	C32	3	1958	1972	0.3	−0.2	0.8	
				1972	1991	−3.2	−3.5	−2.9	^*^
				1991	1997	0.7	−1.3	2.8	
				1997	2018	−3.9	−4.2	−3.7	^*^
	Skin	C43–C44	2	1958	1974	−1.3	−2.0	−0.5	^*^
				1974	1987	−4.8	−5.8	−3.7	^*^
				1987	2018	0.0	−0.3	0.2	
	Brain, nervous system	C70–C72	4	1958	1970	1.3	−0.3	2.9	
				1970	1980	5.7	4.0	7.5	^*^
				1980	1996	1.6	1.1	2.2	^*^
				1996	2007	−0.3	−1.2	0.6	
				2007	2018	3.3	2.6	4.1	^*^
	Multiple myeloma	C88–C90	3	1958	1968	12.8	9.8	15.8	^*^
				1968	1980	6.3	5.0	7.7	^*^
				1980	2000	1.7	1.4	2.1	^*^
				2000	2018	−2.0	−2.3	−1.8	^*^
	Leukemia	C91–C95	4	1958	1976	2.3	2.1	2.6	^*^
				1976	1987	1.4	1.0	1.9	^*^
				1987	1991	−1.9	−4.6	0.8	
				1991	1999	−0.2	−0.9	0.5	
				1999	2018	−1.1	−1.2	−0.9	^*^

ICD-10, International Classification of Diseases, 10th revision.

^*^Annual % change was statistically significantly different from zero (*P* < 0.05).

**Table 4B.** Results of joinpoint regression analysis on the trends in site-specific cancer mortality: national data (1958–2018); Female

Cancer site		ICD-10	Number of joinpoints	Line segment		Annual % change	95% confidence interval		
	
				Start	End		Lower	Upper	
Major									
	Stomach	C16	4	1958	1970	−0.8	−1.0	−0.6	^*^
				1970	1979	−3.2	−3.5	−2.9	^*^
				1979	1991	−4.4	−4.6	−4.2	^*^
				1991	1999	−3.3	−3.8	−2.9	^*^
				1999	2018	−3.9	−4.0	−3.8	^*^
	Colon	C18	5	1958	1982	3.4	3.3	3.5	^*^
				1982	1993	2.3	2.0	2.6	^*^
				1993	2004	−0.4	−0.7	−0.2	^*^
				2004	2008	−2.3	−3.7	−0.9	^*^
				2008	2014	0.4	−0.2	1.0	
				2014	2018	−0.9	−1.8	0.0	
	Rectum	C19–C20	1	1958	1974	1.3	1.0	1.6	^*^
				1974	2018	−1.3	−1.3	−1.2	^*^
	Colon/rectum	C18–C20	5	1958	1974	2.3	2.1	2.6	^*^
				1974	1992	1.1	1.0	1.3	^*^
				1992	2004	−0.5	−0.7	−0.3	^*^
				2004	2009	−2.1	−3.0	−1.2	^*^
				2009	2014	0.4	−0.5	1.3	
				2014	2018	−1.1	−2.0	−0.2	^*^
	Liver	C22	4	1958	1975	−2.4	−2.6	−2.1	^*^
				1975	1989	0.2	−0.1	0.5	
				1989	1999	1.4	0.9	1.8	^*^
				1999	2008	−2.9	−3.4	−2.5	^*^
				2008	2018	−5.7	−6.1	−5.4	^*^
	Pancreas	C25	5	1958	1967	6.4	5.5	7.4	^*^
				1967	1988	2.8	2.7	3.0	^*^
				1988	1994	−0.4	−1.3	0.5	
				1994	2006	0.6	0.4	0.8	^*^
				2006	2011	2.1	1.1	3.1	^*^
				2011	2018	0.7	0.4	1.1	^*^
	Lung, trachea	C33–C34	5	1958	1963	6.5	4.2	8.8	^*^
				1963	1984	3.1	2.9	3.3	^*^
				1984	1998	0.9	0.7	1.1	^*^
				1998	2003	−1.8	−2.8	−0.7	^*^
				2003	2014	−0.2	−0.4	0.1	
				2014	2018	−2.3	−3.3	−1.4	^*^
	Breast	C50	5	1958	1964	−0.5	−2.1	1.1	
				1964	1978	2.3	1.9	2.8	^*^
				1978	1990	1.5	1.1	1.9	^*^
				1990	1997	3.2	2.4	4.0	^*^
				1997	2006	1.4	0.9	1.8	^*^
				2006	2018	0.4	0.2	0.6	^*^
	Uterus	C53–C55	5	1958	1969	−3.1	−3.3	−2.9	^*^
				1969	1979	−4.4	−4.8	−4.1	^*^
				1979	1987	−5.4	−6.0	−4.9	^*^
				1987	1993	−3.1	−4.1	−2.1	^*^
				1993	2007	−0.3	−0.6	−0.1	^*^
				2007	2018	1.0	0.7	1.3	^*^
	Cervix uteri	C53	4	1958	1965	−1.9	−3.3	−0.5	^*^
				1965	1973	−5.1	−6.5	−3.8	^*^
				1973	1978	1.0	−2.4	4.6	
				1978	1989	−2.4	−3.2	−1.6	^*^
				1989	2018	0.4	0.3	0.6	^*^
	Corpus uteri	C54	3	1958	1966	−6.2	−8.1	−4.3	^*^
				1966	1972	−14.3	−19.1	−9.3	^*^
				1972	1996	4.3	3.8	4.7	^*^
				1996	2018	3.0	2.7	3.3	^*^
Sub-major									
	Esophagus	C15	2	1958	1969	−0.1	−0.7	0.5	
				1969	1989	−3.9	−4.1	−3.6	^*^
				1989	2018	−0.7	−0.9	−0.6	^*^
	Gallbladder and bile ducts	C23–C24	4	1958	1963	12.0	9.0	15.1	^*^
				1963	1972	6.1	5.1	7.0	^*^
				1972	1985	4.1	3.7	4.4	^*^
				1985	1992	0.1	−0.5	0.8	
				1992	2018	−3.1	−3.1	−3.0	^*^
	Ovary	C56	4	1958	1981	3.9	3.7	4.1	^*^
				1981	1996	1.3	1.1	1.6	^*^
				1996	2000	−2.6	−5.1	0.0	^*^
				2000	2011	−0.2	−0.6	0.2	
				2011	2018	−1.1	−1.8	−0.4	^*^
	Urinary bladder	C67	2	1958	1969	1.6	0.6	2.6	^*^
				1969	1990	−1.7	−2.0	−1.4	^*^
				1990	2018	−0.2	−0.3	0.0	^*^
	Kidney and other urinary organs (except bladder)	C64–C66 C68	3	1958	1976	1.5	0.9	2.1	^*^
				1976	1986	3.8	2.6	5.1	^*^
				1986	2006	1.5	1.2	1.7	^*^
				2006	2018	0.0	−0.4	0.4	
	Thyroid	C73	2	1958	1977	1.9	1.5	2.4	^*^
				1977	2003	−1.0	−1.2	−0.8	^*^
				2003	2018	−1.8	−2.1	−1.4	^*^
	Malignant lymphoma	C81–C85 C96	3	1958	1981	2.5	2.3	2.7	^*^
				1981	2001	0.9	0.7	1.1	^*^
				2001	2006	−2.2	−3.8	−0.5	^*^
				2006	2018	0.0	−0.3	0.3	
Others									
	Oral cavity and pharynx	C00–C14	3	1958	1979	0.6	0.2	1.1	^*^
				1979	1991	−0.8	−1.7	0.0	
				1991	1998	5.2	3.3	7.1	^*^
				1998	2018	−0.5	−0.8	−0.3	^*^
	Larynx	C32	2	1958	1976	−4.3	−5.0	−3.5	^*^
				1976	1986	−9.0	−11.3	−6.6	^*^
				1986	2018	−3.5	−4.0	−3.0	^*^
	Skin	C43–C44	3	1958	1973	−1.8	−2.8	−0.8	^*^
				1973	1988	−5.1	−6.1	−4.1	^*^
				1988	2012	0.8	0.3	1.3	^*^
				2012	2018	−2.7	−5.9	0.7	
	Brain, nervous system	C70–C72	4	1958	1971	1.8	−0.1	3.7	
				1971	1978	7.3	2.8	12.1	^*^
				1978	1998	1.5	1.0	2.1	^*^
				1998	2007	−0.6	−2.4	1.3	
				2007	2018	3.2	2.1	4.3	^*^
	Multiple myeloma	C88–C90	4	1958	1968	14.8	11.9	17.9	^*^
				1968	1983	5.6	4.8	6.4	^*^
				1983	1997	1.5	1.0	1.9	^*^
				1997	2005	−0.2	−1.2	0.9	
				2005	2018	−2.6	−3.0	−2.2	^*^
	Leukemia	C91–C95	2	1958	1967	2.9	2.1	3.8	^*^
				1967	1987	0.6	0.4	0.8	^*^
				1987	2018	−1.5	−1.6	−1.4	^*^

ICD-10, International Classification of Diseases, 10th revision.

^*^Annual % change was statistically significantly different from zero (*P* < 0.05).

**Figure 4.**
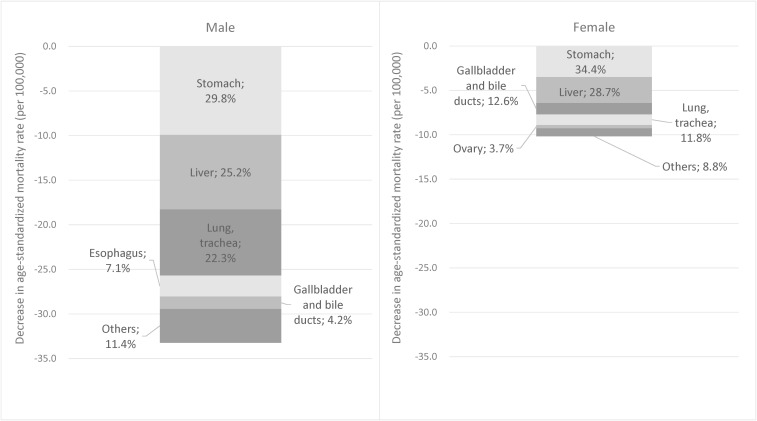
Contribution of cancer sites to the decrease in mortality in the most recent 10 years (2009–2018).

Figure 4 shows the contribution of specific cancer sites to the significant decrease in all-cancer mortality in the most recent 10 years (2009–2018). For males, stomach cancer accounted for 29.8% of the decrease of all-cancer incidence, followed by liver (25.2%) and lung (22.3%). These three sites accounted for 77.3% of the all-cancer decrease. Esophagus, and gallbladder and bile ducts accounted for less than 10% (7.1% and 4.2%, respectively). For females also, stomach, liver, and lung accounted for nearly 75% of the all-cancer decrease (34.4%, 28.7%, and 11.8%, respectively). Unlike the result in males, however, the contribution of gallbladder and bile ducts was slightly larger than that of lung (12.6%), while ovary contributed 3.7%.

The contributions of cancer sites to the significant changes in all-cancer incidence and mortality under 75 years old are shown in eFigure 1 and eFigure 2, respectively. The largest contributions of prostate in males and breast in females were the same as in the results for all ages. There was no significant decrease in all-cancer incidence when age was restricted to under 75 years old. The large contributions of stomach, liver, lung, and gallbladder and bile ducts to the recent reduction in all-cancer mortality were also the same as the result of all ages.

Figure 5A shows the trends in incidence and mortality of all cancers combined. There was a marked divergence between the trends in incidence and mortality in both males and females. For males, the divergence became wider after the late 1990s due to the decrease in mortality. For females, the gap between incidence and mortality widened constantly. Figure 5B and Figure 5C show the trends in incidence and mortality of major and sub-major cancer sites, respectively. Similar to all cancers combined, a divergence between incidence and mortality was a common feature observed in almost all cancer sites. The results of cancers other than major and sub-major sites are shown in eFigure 3.

**Figure 5A.**
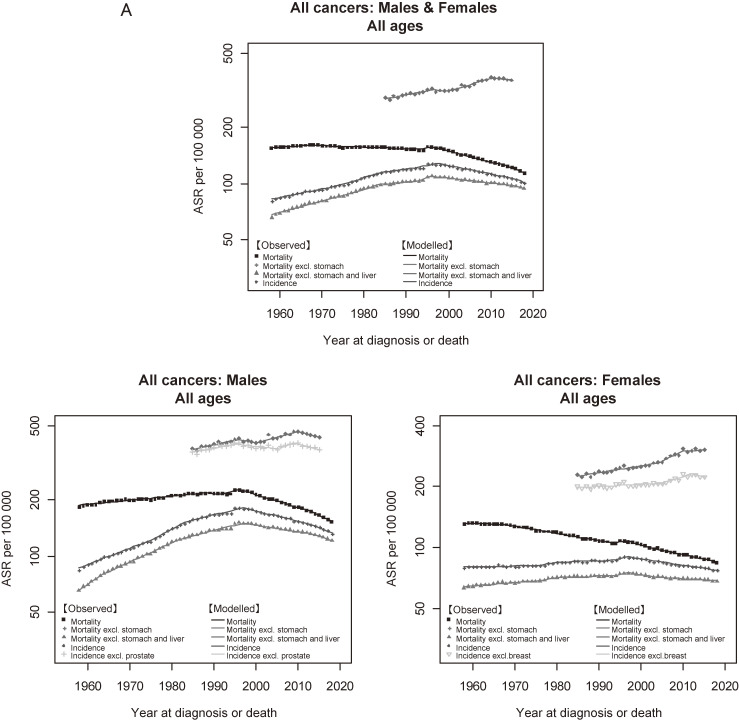
Observed and modelled trends in cancer incidence (1985–2015) and mortality (1958–2018) rates: All cancers combined. ^a^Incidence: data from Yamagata, Fukui, and Nagasaki Prefectures, Mortality: national data. ^b^Standardized to the Japanese model population in 1985.

**Figure 5B.**
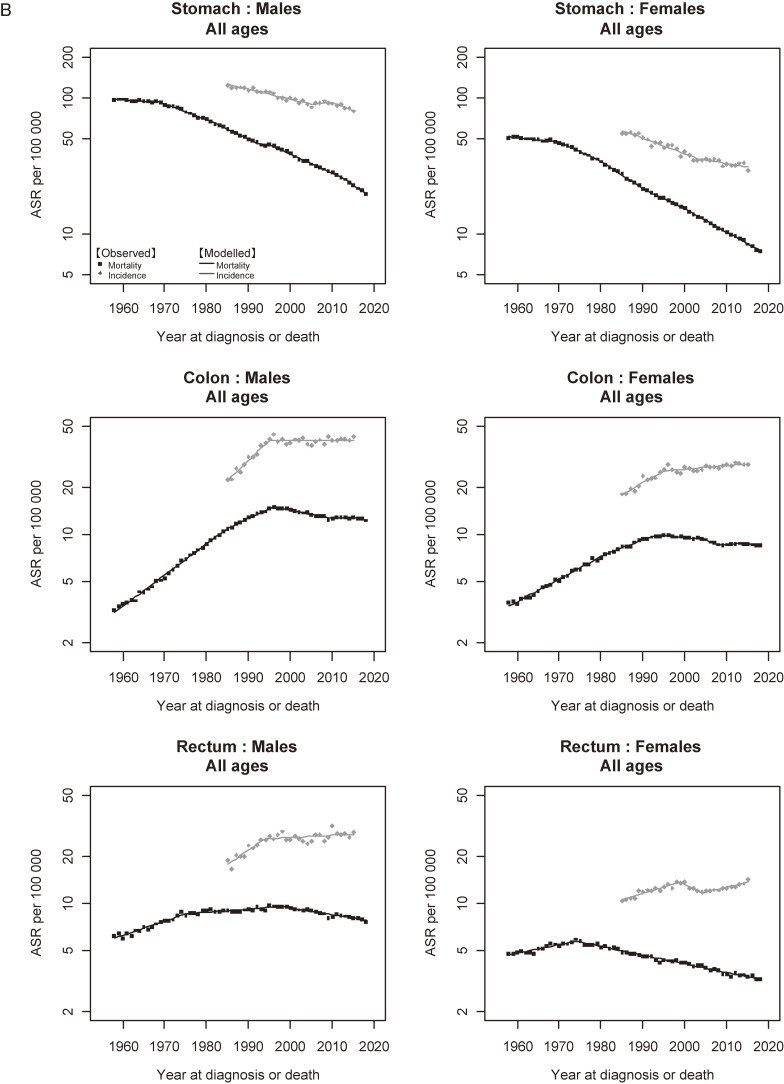

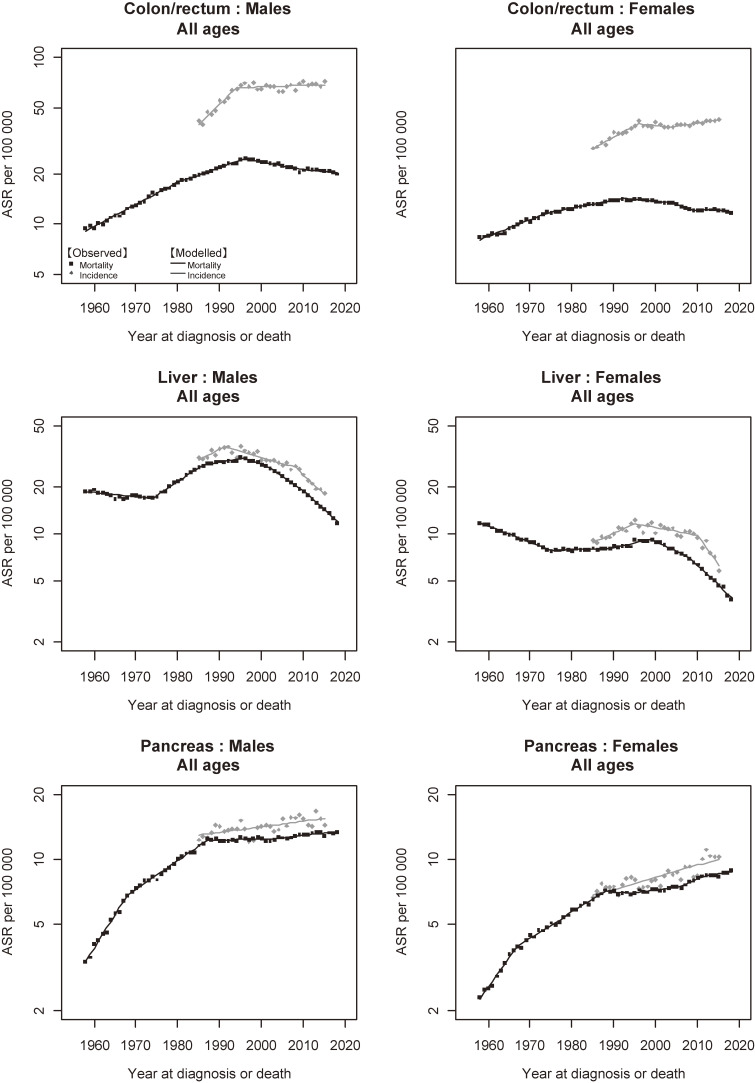

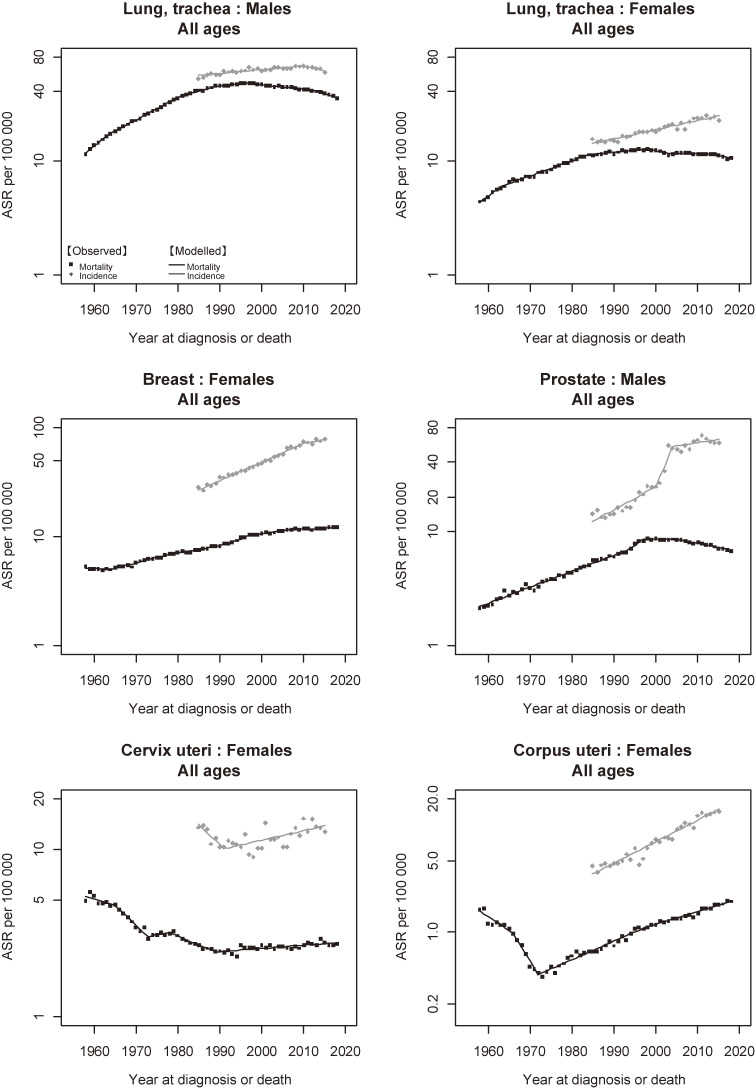
Observed and modelled trends in cancer incidence (1985–2015) and mortality (1958–2018) rates: Major sites. ^a^Incidence: data from Yamagata, Fukui, and Nagasaki Prefectures, Mortality: national data. ^b^Standardized to the Japanese model population in 1985.

**Figure 5C.**
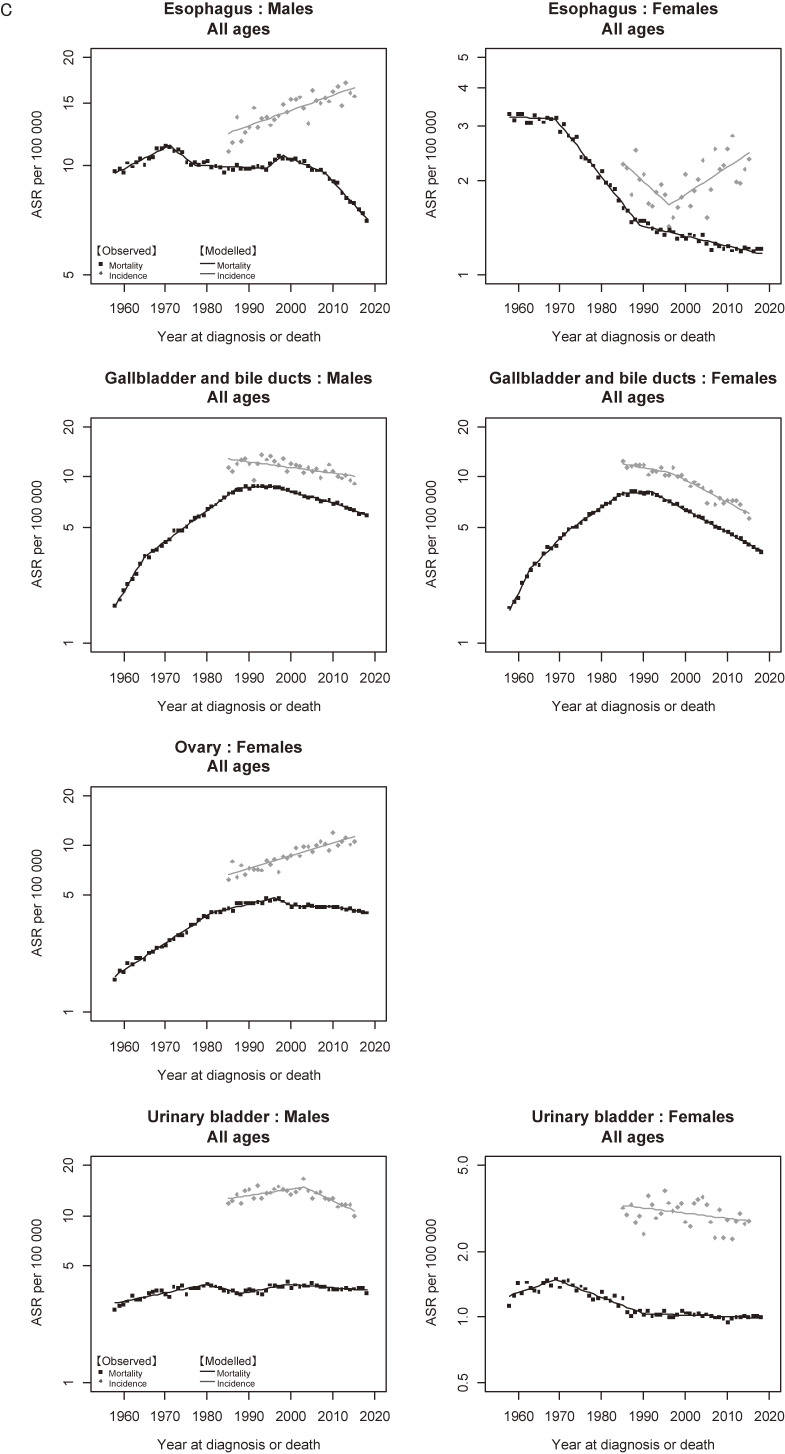

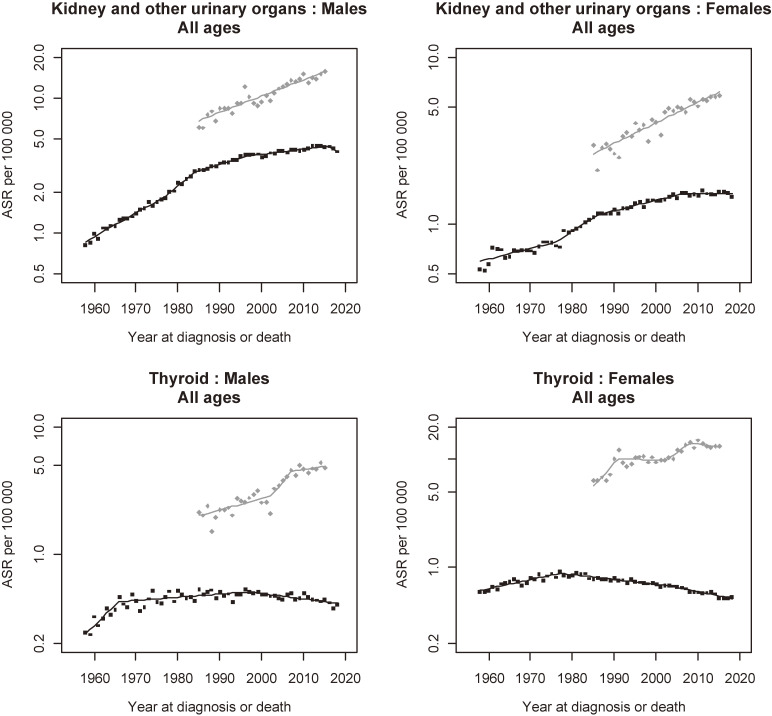

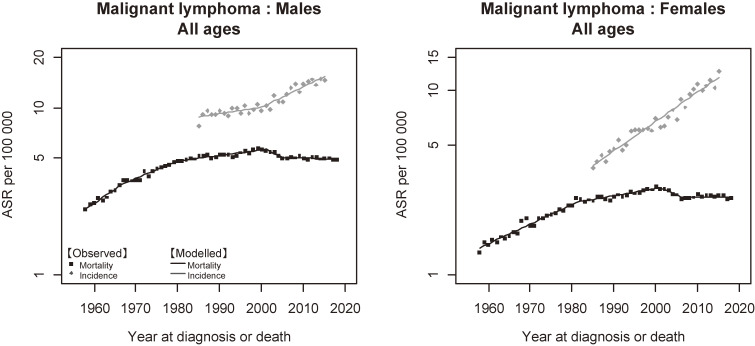
Observed and modelled trends in cancer incidence (1985–2015) and mortality (1958–2018) rates: Sub-major sites. ^a^Incidence: data from Yamagata, Fukui, and Nagasaki Prefectures, Mortality: national data. ^b^Standardized to the Japanese model population in 1985.

Table 5 summarizes the description of observed trends in incidence and mortality and potential interpretations for each cancer site. Decreases in exposure to major risk factor such as infectious agents and tobacco smoking were considered to be associated with the decreases in incidence and/or mortality of related cancers (stomach, liver, lung, and urinary bladder). Effects of the introduction and dissemination of cancer screening were considered to have been reflected in the trends in incidence and/or mortality of several cancers (stomach, colon/rectum, female breast, and prostate), among which the increase in prostate cancer incidence was most remarkable. Improvements in diagnostic measures and treatments were common factors associated with the divergence of incidence and mortality. Potential overdiagnosis was considered to be included in the increase in incidence of prostate and thyroid cancers.

**Table 5.** Summary of the trends in age-standardized incidence and mortality for major and sub-major cancer sites

	Cancer site	ICD-10	Sex	Incidence	Mortality	Possible interpretation	

						Phenomenon [references, if any]	Related factors [references, if any]
Major	Stomach	C16	Male	Decreased continuously (1985–2005, 2009–2015)	Decreased continuously (1958–1992, 1996–2018)	- Long-term decrease in incidence and mortality - Steeper decrease in mortality than incidence - Acceleration of mortality reduction in males	- Decrease in the prevalence of Helicobacter pylori infection combined with improvements in sanitation, diet (reduced salt intake), and food preservation techniques[11,32,33] - Early detection and improvements in prognosis,[11] especially after 2000[70] - Coverage of H. pylori eradication in 2013[35] (not consistent in females or incidence)
			Female	Decreased continuously (1985–2015)	Decreased continuously (1958–2018)		
	
	Colon/rectum	C18–C20	Male	Increased until 1990s (1985–1994), levelled off thereafter (1994–2015)	Increased until 1990s (1958–1996), decreased thereafter (1996–2018)	- Increase and peaking out of incidence and mortality - Levelling of incidence - Recent slight decrease in mortality[11,70]	- Spread of Westernized lifestyles in 1970s[3,11,74,75] - Introduction of organized screening (fecal occult blood test) in 1992[11,74] - Improvements in prognosis[11,70]
			Female	Increased until 1990s (1985–1996), slowly increased thereafter (2003–2015)	Increased until 1990s (1958–1992), intermittently decreased thereafter (1992–2009, 2014–2018)		
	
	Liver	C22	Male	Increased until 1990s (1985–1991), decreased thereafter (1991–2015) (accelerated in 2008)	Increased until 1990s (1974–1996), decreased thereafter (1996–2018) (accelerated in 2010)	- Divergence between incidence and mortality since 1980s - Decrease in incidence and mortality since 1990s - Acceleration of the decrease in incidence and mortality[11] - Decrease in mortality	- Improvements in differential diagnosis by the introduction of imaging techniques and biomarkers - Long-term decrease in in the prevalence of hepatitis virus (mainly HCV)[11,37] - Therapeutic improvements including pegylated interferon and direct acting antivirals[11] - Improvement in survival after 2000 (Liver cancer[70])
			Female	Increased until 1990s (1985–1995), decreased thereafter (1995–2015) (accelerated in 2010)	Decreased (1958–1975) and increased (1989–1999) until 1990s, decreased thereafter (1999–2018) (accelerated in 2008)		
	
	Pancreas	C25	Male	Increased continuously (1985–2015)	Increased continuously (1958–1987, 2002–2018) (slowed down in 1987)	- Increase in incidence and mortality - Divergence between incidence and mortality	- Increase in risk factors such as type 2 diabetes[56] - Improvements in diagnostic measures such as computed tomography imaging and biopsy for histologic confirmation[57] - Improvement in survival after 2000[70]
			Female	Increased continuously (1985–2015)	Increased continuously (1958–1988, 1994–2018) (slowed down in 1988)		
	
	Lung, trachea	C33–C34	Male	Increased until 2010s (1985–2010), decreased thereafter (2010–2015)	Increased until 1990s (1958–1996), decreased thereafter (1996–2018)	- Decrease in mortality and incidence of squamous and small-cell lung cancer after 1990s[46–48] - Increase in incidence (especially adenocarcinoma)[46–48] - Decrease in mortality	- Decrease in smoking prevalence[46–48] - Shift from non-filtered to filtered cigarettes[48] - Improvement in diagnostic measures such as CT[46,47] - Improvement in prognosis for patients with chemotherapy[76] - Improvement in survival after 2005[70]
			Female	Increased continuously (1985–2015)	Increased until 1990s (1958–1998), decreased intermittently thereafter (1998–2003, 2014–2018)		
	
	Breast	C50	Female	Increased until 2010s (1985–2010), levelled off thereafter (2010–2015)	Increased continuously but gradually slowed down (1964–2018)	- Long-term Increase in incidence and mortality - Slowing down of mortality and Increase in the incidence of carcinoma in situ[11,31] - Increase in local cases and decrease in regional cases[31] - Divergence between incidence and mortality	- Effect of reproductive factors (younger menarche, older age at birth, lower parity)[11,29,30] - Dissemination of screening[11,31] - Improvement in diagnostic technology[31] - Improvement in survival from 1993 to 2006[71]
	
	Cervix uteri	C53	Female	Decreased until 1990s (1985–1991), increased thereafter (1991–2015)	Decreased intermittently until 1980s (1958–1973, 1978–1989), increased thereafter (1989–2018)	- Increase in incidence and mortality - Increase in mortality - Divergence between incidence and mortality	- Increasing prevalence of human papillomavirus (HPV) infection among young women[78] - Poor uptake of screening[11] - Improvement in prognosis after 2002, especially in cases of “localized” and “adjacent organs”[79] or stage III[77] likely due to the introduction of concurrent chemotherapy and radiation and dissemination of clinical guidelines
	
	Corpus uteri	C54	Female	Increased continuously (1985–2015)	Decreased until 1970s (1958–1972), increased continuously thereafter (1972–2018)	- Increase in incidence and mortality - Divergence between incidence and mortality	- Long-term effect of reproductive factors (older and fewer births)[11] - Improvement in prognosis for patients of stage I or II receiving adjuvant chemotherapy[80]
	
	Prostate	C61	Male	Increased continuously (1985–2015) (accelerated between 2000–2004)	Increased until 1990s (1958–1997), decreased from 2000s (2005–2018)	- Rapid increase in incidence in the early 2000s prominent in localized cases[26] - Slowing down of the increase in prostate cancer incidence in 2004 - Sharp increase in incidence in the absence of a clear change in mortality - Divergence between incidence and mortality - Levelling of and decrease in mortality since 1990s	- Spread of prostate-specific antigen (PSA) screening[26] - Convergence of the spread of PSA screening - Potential overdiagnosis[11,58,59] - Improvement in survival after 2000[70] - Improvement of prognosis since 1990s by the introduction of hormone therapy[63] and other refinements in treatment and disease management[64]

Sub-major	Esophagus	C15	Male	Increased continuously (1985–2015)	Increased (1958–1971, 1994–1998), decreased (1971–1977) intermittently until 1990s, decreased thereafter (1998–2018)	- Long-term increase in incidence in males - Decrease in mortality - Divergence between incidence and mortality	- Increase in incidence of gastroesophageal reflux disease associated with decrease in the prevalence of Helicobacter pylori infection[36] - Expansion of endoscopic procedure of upper gastrointestinal tract[82] and advances in treatment[81] - Improvement in survival after 2000[70]
			Female	Decreased until 1990s (1985–1996), increased thereafter (1996–2015)	Decreased continuously (1969–2018) (slowed down in 1989)		
	
	Gallbladder and bile ducts	C23–C24	Male	Decreased continuously (1985–2015)	Increased until 1980s (1958–1987), decreased from 1990s (1995–2018)	- Long-term decrease in incidence and mortality -Similarity of the incidence and mortality trends to liver cancer	- Changes in risk factors such as gallstones, body fatness or obesity, and chronic infections [41–43] - Control over communicable diseases[44] - Overlapping of risk factors and misclassification between intra- and extra-hepatic cholangiocarcinoma[45]
			Female	Decreased continuously (1985–2015)	Increased until 1980s (1958–1985), decreased from 1990s (1992–2018)		
	
	Ovary	C56	Female	Increased continuously (1985–2015)	Increased until 1990s (1958–1996), decreased intermittently thereafter (1996–2000, 2011–2018)	- Long-term increase in incidence - Divergence between incidence and mortality	- Changes in reproductive factors (eg younger menarche, older age at birth, lower parity[29,30] and increase in endometriosis) - Improvement of prognosis since 1990s by the introduction of new chemotherapies,[83] molecular target drugs, and also aggressive debulking surgery[84] - Improvement in survival after 2000[70]
	
	Urinary bladder	C67	Male	Increased until 2000s (1985–2003), decreased thereafter (2003–2015)	Increased (1958–1980, 1988–1999), decreased (1980–1988, 1999–2018) intermittently	- Decrease in incidence in males after 1990s - Absence of steep decrease in mortality	- Decrease in prevalence of tobacco smoking[86] - Improvement in survival in 1995–2004 predominantly observed for cases with “regional” stage,[85] which could have been masked due to the predominance of “localized” stage[25,85]
			Female	Decreased continuously (1985–2015)	Decreased continuously (1969–2018) (slowed down in 1990)		
	
	Kidney and other urinary organs (except bladder)	C64–C66 C68	Male	Increased continuously (1985–2015)	Increased continuously until 2010s (1958–2016), decreased thereafter (2016–2018)	- Long-term increase in incidence and mortality - Long-term increase in incidence - Divergence between incidence and mortality	- Tobacco smoking[49,50] - Changes in other potential risk factors (eg hypertension, diabetes)[90,91] - Improvement of diagnostic imaging techniques[88] -Improvement in therapy, surgery, and noninvasive tumor imaging in renal cell carcinoma since the late 1990s[87,89]
			Female	Increased continuously (1985–2015)	Increased continuously until 2000s (1958–2006), levelled off thereafter (2006–2018)		
	
	Thyroid	C73	Male	Increased until 2000s (1985–2007), levelled off thereafter (2007–2015)	Increased until 1990s (1958–1997), decreased thereafter (1997–2018)	- Increases in incidence of small papillary lesions - Divergence between incidence and mortality	- Increased medical surveillance of thyroid nodules and symptoms combined with improvement of diagnostic imaging techniques including ultrasonography, leading to a potential overdiagnosis[60,61] - Improved diagnosis, treatment, and disease management[60,61]
			Female	Increased intermittently until 2000s (1985–1991, 2002–2008), levelled off thereafter (2008–2015)	Increased until 1970s (1958–1977), decreased thereafter (1977–2018)		
	
	Malignant lymphoma	C81–C85 C96	Male	Increased continuously (1985–2015) (accelerated in 2000)	Increased until 2000s (1958–2001), decreased temporarily (2001–2005), levelled off thereafter (2005–2018)	- Long-term increases in incidence and mortality - Divergence between incidence and mortality	- Westernization of lifestyles[66] - Improvements in diagnosis and coding of registry data[65,66] - Improvement in prognosis[65], consistent with increase in survival after 2000[70]
			Female	Increased continuously (1985–2015)	Increased until 2000s (1958–2001), decreased temporarily (2001–2006), levelled off thereafter (2006–2018)		

ICD-10, International Classification of Diseases, 10th revision.

## DISCUSSION

This study analyzed the trends in cancer incidence and mortality in Japan with updated representative datasets. A notable finding was that the ASR of all-cancer incidence started to significantly decrease in males and level off in females in 2010, after a long-term intermittent increase.^11^^,^^12^^,^^25^ The leading cancer sites that contributed to the past long-term increase were prostate in males and breast in females, but these slowed down or levelled off during the first decade of 2000s. For males, the main contributing cancer sites to this significant decrease were stomach, liver and lung cancers.

The ASR of prostate cancer incidence dramatically increased between 2000 and 2004 (APC 22.4%) and slowed down thereafter (APC 1.3% in 2004–2015). As summarized in Table 5, this rapid increase in the early 2000s was prominent for localized cancer and suggested the contribution of the spread of prostate-specific antigen (PSA) screening.^26^ Indeed, PSA screening increased almost threefold in 2003 both in terms of the number of participants and the number of municipalities that adopted it as an organizational screening program, and subsequently shifted to a slow increase.^27^^,^^28^

The levelling off of ASR of female breast cancer incidence after 2010 observed in the present study is an unprecedented phenomenon.^11^^–^^13^ The mortality of female breast cancer^12^ also slowed a slowing down of its increasing trend. Long-term increase in incidence as well as mortality can be interpreted as the effect of changes in reproductive factors in Japanese females (Table 5).^11^^,^^29^^,^^30^ This effect might be converging in breast cancer, but cancers of the corpus uteri and ovary, which share common reproductive risk factors, continued to increase in incidence. The participation rate of female breast cancer screening (mammography) has been increasing in Japan, and early-stage cancer and carcinoma in situ of the breast was reported to have increased in a study using a prefectural cancer registry.^31^ Together with the slowing down of the increasing trend in mortality observed in the present study, these changes in trends could have partially reflected the dissemination of breast cancer screening.^11^

The significant decrease in ASR of all-cancer incidence in males after 2010 was accounted for by stomach, liver, and lung cancers. These cancer sites also contributed to the decrease in all-cancer mortality in both males and females (77.3% and 74.9%, respectively; Figure 4). Stomach cancer consistently decreased during the whole observation period with regard to both incidence and mortality, which can be interpreted to be the result of dramatic reduction of Helicobacter pylori (H. pylori) infection, combined with improvements in sanitation, diet (reduced salt intake), and food preservation techniques (Table 5).^11^^,^^32^^,^^33^ A study using data from a hospital-based registry in 2007–2015 showed a decrease in the proportion of cancer in the pylorus, the main subsite of H. pylori-induced cancer.^34^ A related factor is the eradication of H. pylori, which was covered by the universal health insurance in Japan in 2013 as part of the treatment for chronic gastritis as well as for gastric and duodenal ulcer. Indeed, the reported number of eradications doubled after this extension of coverage.^35^ Although we observed an acceleration in the decrease in stomach cancer mortality in males (APC −3.2% in 1996–2012, −4.6% in 2012–2018; Table 4A), this was not consistent for incidence nor in females. Thus, the effect of H. pylori eradication is not clear at the population level.

Evidence of a relation between the absence of H. pylori infection and risk of adenocarcinoma of the esophagus is accumulating.^36^ The long-term decrease in the prevalence of H. pylori in Japan may be related to the increase in esophageal cancer incidence observed in the present study, through the pathway from gastroesophageal reflux disease to the occurrence of esophageal cancer (Table 5).

Liver cancer is another site that showed a dramatic decrease in incidence and mortality. As discussed in previous literature, the long-term decrease in liver cancer in Japan is mainly due to the decrease in the prevalence of hepatitis C virus (HCV).^11^^,^^13^^,^^37^ The observed acceleration both in incidence and mortality in 2008 or 2010 (Table 2 and Table 4) can be interpreted as a reflection of therapeutic improvements made in the early 2000s such as pegylated interferon in 2004 and a protease inhibitor (Telaprevire) in 2011.^11^^,^^38^^–^^40^ Improvements in differential diagnosis could have also affected the divergence between incidence and mortality since 1980s (Table 5).

Cancer of the gallbladder and bile ducts had a similar pattern of trends to that of liver cancer. Chronic infections have been proposed as one of the risk factors for gallbladder cancer as well as gallstones and obesity (Table 5).^41^^–^^43^ Control over communicable diseases could have resulted in the reduction of incidence rate of gallbladder cancer.^44^ Regarding cholangiocarcinoma (CCA), overlapping of risk factors and misclassification between intra- and extra-hepatic CCA^45^ might explain the similarity to the trends in liver cancer and cancer of the combined category of gallbladder and bile ducts.

The decrease in lung cancer incidence in males was a phenomenon that had never been observed in previous literature.^11^^–^^13^ Trends in lung cancer incidence in Osaka by histological type revealed that the ASR of adenocarcinoma continuously increased, whereas those of squamous and small-cell carcinomas decreased from 1990s, which was interpreted to be the result of the spread of diagnostic use of computed tomography and the decreasing trend in smoking prevalence, respectively.^46^^,^^47^ Another possibility is the shift from nonfilter to filtered cigarettes in the consumption of tobacco products in Japan (Table 5), which may be more influential because the increase in adenocarcinoma was observed even before the introduction of major diagnostic advances.^48^ The decrease in overall lung cancer incidence observed in the present study could have reflected the predominant effect of declining smoking prevalence, albeit that analysis stratified by histological type is needed to clarify this possibility. Cancers of the kidney and urinary tracts are also strongly related to tobacco smoking,^49^^,^^50^ but no similarity was found between the trends in these cancers and smoking prevalence except for bladder cancer incidence in males (Table 5).

An important feature of our results is that a decrease in incidence was not observed for colorectal cancer, which can be prevented by organizational screening. The ASR of colorectal cancer has been significantly decreasing in many countries.^1^^–^^3^^,^^5^ Using simulation modeling techniques, one study revealed that the reduction in colorectal cancer in the United States was a combined effect of cancer control measures for prevention and screening.^6^ Cervical cancer also showed a sharp contrast; the ASR of this cancer has been consistently decreasing overseas, including the Republic of Korea,^1^^,^^2^^,^^5^ whereas the present study showed a significant increase in incidence and mortality, just as was observed in our previous analysis.^11^^–^^13^ The increase in mortality of cervical cancer (cancer of the corpus uteri as well) in Japan should be interpreted with caution because it could have included the shift from cancer of the ‘uterus, not otherwise specified (NOS)’. However, the proportion of NOS had been stable since the late 1990s, and the increase was also observed in incidence.^11^^,^^13^ Cervical cancer can be prevented by a combination of organizational screening and human papillomavirus (HPV) vaccination.^7^^,^^9^^,^^51^^,^^52^ In Japan, the national HPV vaccination program has been substantially halted by the fear of potential adverse effects.^53^^,^^54^ A simulation modeling study demonstrated that rapid restoration of vaccination coverage and catch-up for missed cohorts could avoid approximately 50,000 cervical cancer cases in 50 years.^55^ Realizing a reduction in colorectal and cervical cancer incidence by promoting the primary and secondary preventive measures is a major challenge for Japan.

Pancreatic cancer was another example that showed a long-term increase both in incidence and mortality. Increase in risk factors, such as type 2 diabetes, may be related to the increase in incidence^56^ and mortality as well. Improvements in diagnostic measures and biopsy for histologic confirmation have also been proposed as underlying factors of the increasing trend in earlier years.^57^

Monitoring cancer incidence trends is useful in examining the possibility of overdiagnosis at a population level. In the United States, prostate, female breast, skin, kidney, thyroid, and lung cancers have been listed as examples of potential overdiagnosis, characterized as a sharp increase in incidence in the absence of a clear change in mortality.^58^ In the present study, this typical pattern seemed to be observed for prostate and thyroid cancers (Figure 5 and Table 5). A common background factor of these cancers is the availability of non-invasive tests (ie, PSA for prostate cancer and ultrasonography for thyroid cancer).^11^^,^^26^^,^^58^^–^^61^ In addition to this descriptive approach, empirical or modelling approaches that compare screened and unscreened (or tested and untested) populations are needed to quantitively examine overdiagnosis.^62^ Despite the possibility of overdiagnosis, both prostate and thyroid cancers showed significant decreases in mortality. Refinements in diagnosis, treatment and disease management could have contributed to those trends in mortality.^60^^,^^61^^,^^63^^,^^64^ Malignant lymphoma showed a pattern of sharp increase in incidence and no clear change in mortality, but changes in lifestyle and improvements in diagnosis and prognosis, as well as in coding of registry data, have been proposed as underlying factors.^65^^,^^66^

Some of the reduction in cancer mortality observed in the present and previous studies^11^^–^^13^ likely reflects improvements in the prognosis of cancer patients. This effect can be seen in the divergence between trends in incidence and mortality (Figure 5), which is consistent with the evidence of improved diagnosis, treatment, and disease management cited in Table 5. Indeed, several studies on hematological cancers showed that the introduction of a new drug or treatment was associated with a reduction in mortality at the national level.^67^^–^^69^ Studies using population-based cancer registries have also reported increases in survival rates that can be interpreted as a reflection of improvements in treatment.^46^^,^^70^^–^^73^ Our study group is planning to update these reports using the most recent MCIJ dataset (patients diagnosed in 1993–2015).

A strength of the present study is the representativeness of the data. Mortality data were from the national vital statistics and based on a complete mandatory reporting system. Incidence data were from three prefectures, but the representativeness of the data in terms of secular trends has been validated.^12^^,^^19^

One of the limitations of the present study is that the trends in cancer incidence might have been affected by an improvement in the completeness and data quality of prefectural cancer registries. Indeed, even in the three present prefectures that have long-term high-quality data, there was a slight increase in completeness and quality indices during the first decade of the 2000s (eFigure 4). The observed increases in incidence in this period might therefore reflect an improvement in data completeness. A second limitation is that our analysis was only descriptive. Furthermore, the cancers we analyzed were not grouped into clinically relevant subtypes. As stated above, further research is required to clarify factors underlying the observed cancer trends, such as analyses according to clinical stage or histological type and modelling approaches.

In conclusion, this analysis of cancer trends in Japan revealed that the ASR of all-cancer incidence started to decrease significantly in males and level off in females in 2010, after a long-term intermittent increase. The convergence of an increase in all-cancer incidence was mainly due to the slowing down of prostate and breast cancers in males and females, respectively. The ASR of all-cancer mortality continued to decrease, the main contributing cancer sites of which were still stomach, liver, and male lung.

## APPENDIX A. SUPPLEMENTARY DATA

eFigure 1. Contribution of cancer sites to the changes in incidence; Under 75 years old

eFigure 2. Contribution of cancer sites to the decrease in mortality in recent 10 years (2009–2018); Under 75 years old

eFigure 3. Observed and modelled trends in cancer incidence (1985–2015) and mortality (1958–2018) rates: Cancers other than major and sub-major sites.

eFigure 4. Annual trends in the quality indexes of cancer incidence data from three Prefectures (1985–2015)

eTable 1. Results of joinpoint regression analysis on the trends in all-cancer incidence: data from three prefectures (1985–2015): Under 75 years old^a^

eTable 2A. Results of joinpoint regression analysis on the trends in site-specific cancer incidence: data from three prefectures (1985–2015); Male, including cis^a^

eTable 2B. Results of joinpoint regression analysis on the trends in site-specific cancer incidence: data from three prefectures (1985–2015); Female, including CIS

eTable 3. Results of joinpoint regression analysis on the trends in all-cancer mortality: national data (1958–2018): Under 75 years old

## References

[1] Henley SJ, Ward EM, Scott S, . Annual Report to the Nation on the Status of Cancer, part I: National cancer statistics. Cancer. 2020;126(10):2225–2249. 10.1002/cncr.32802 32162336 PMC7299151

[2] *Canadian Cancer Statistics* *2019*. Canadian Cancer Society, [Apr. 1, 2020 accessed]; Available from: http://cancer.ca/Canadian-Cancer-Statistics-2019-EN.

[3] Arnold M, Sierra MS, Laversanne M, Soerjomataram I, Jemal A, Bray F. Global patterns and trends in colorectal cancer incidence and mortality. Gut. 2017;66:683–691. 10.1136/gutjnl-2015-310912 26818619

[4] Dafni U, Tsourti Z, Alatsathianos I. Breast cancer statistics in the European Union: incidence and survival across European countries. Breast Care (Basel). 2019;14:344–353. 10.1159/000503219 31933579 PMC6940474

[5] Hong S, Won YJ, Park YR, Jung KW, Kong HJ, Lee ES; Community of Population-Based Regional Cancer Registries. Cancer statistics in Korea: incidence, mortality, survival, and prevalence in 2017. Cancer Res Treat. 2020;52:335–350. 10.4143/crt.2020.206 32178489 PMC7176962

[6] Edwards BK, Ward E, Kohler BA, . Annual report to the nation on the status of cancer, 1975–2006, featuring colorectal cancer trends and impact of interventions (risk factors, screening, and treatment) to reduce future rates. Cancer. 2010;116:544–573. 10.1002/cncr.24760 19998273 PMC3619726

[7] Guo F, Cofie LE, Berenson AB. Cervical cancer incidence in young U.S. females after human papillomavirus vaccine introduction. Am J Prev Med. 2018;55:197–204. 10.1016/j.amepre.2018.03.013 29859731 PMC6054889

[8] Holford TR, Meza R, Warner KE, . Tobacco control and the reduction in smoking-related premature deaths in the United States, 1964–2012. JAMA. 2014;311:164–171. 10.1001/jama.2013.285112 24399555 PMC4056770

[9] Lee JH, Kim H, Choi H, . Contributions and limitations of National Cervical Cancer Screening Program in Korea: a retrospective observational study. Asian Nurs Res (Korean Soc Nurs Sci). 2018;12:9–16. 10.1016/j.anr.2017.12.002 29463482

[10] Thun M, Peto R, Boreham J, Lopez AD. Stages of the cigarette epidemic on entering its second century. Tob Control. 2012;21:96–101. 10.1136/tobaccocontrol-2011-050294 22345230

[11] JACR Monograph Supplement 2 “Cancers truely on the rise or decline”. Tokyo, Japan: Japanese Association of Cancer Registries; 2016 [in Japanese].

[12] Katanoda K, Hori M, Matsuda T, . An updated report on the trends in cancer incidence and mortality in Japan, 1958–2013. Jpn J Clin Oncol. 2015;45:390–401. 10.1093/jjco/hyv002 25637502

[13] Katanoda K, Matsuda T, Matsuda A, . An updated report of the trends in cancer incidence and mortality in Japan. Jpn J Clin Oncol. 2013;43:492–507. 10.1093/jjco/hyt038 23493744

[14] *Fukui Prefecture Cancer Registry*. Fukui Prefecture, [Nov. 13, 2020 accessed]; Available from: https://www.pref.fukui.lg.jp/doc/kenkou/gantouroku.html [in Japanese].

[15] *Nagasaki Prefecture Cancer Registry Report*. Nagasaki Prefecture, [Nov. 13, 2020 accessed]; Available from: http://gantaisaku.pref.nagasaki.jp/nagasaki_report.html [in Japanese].

[16] *Yamagata Prefecture Cancer Incidence Data Tables*. Yamagata Prefecture, [Nov. 13, 2020 accessed]; Available from: https://www.pref.yamagata.jp/090015/kenfuku/iryo/gan/kankeishamuke/gantouroku/syuukei.html [in Japanese].

[17] Hori M, Matsuda T, Shibata A, Katanoda K, Sobue T, Nishimoto H; Japan Cancer Surveillance Research Group. Cancer incidence and incidence rates in Japan in 2009: a study of 32 population-based cancer registries for the Monitoring of Cancer Incidence in Japan (MCIJ) project. Jpn J Clin Oncol. 2015;45:884–891. 10.1093/jjco/hyv088 26142437

[18] Matsuda T, Sobue T. Recent trends in population-based cancer registries in Japan: the Act on Promotion of Cancer Registries and drastic changes in the historical registry. Int J Clin Oncol. 2015;20:11–20. 10.1007/s10147-014-0765-4 25351534

[19] Katanoda K, Ajiki W, Matsuda T, . Trend analysis of cancer incidence in Japan using data from selected population-based cancer registries. Cancer Sci. 2012;103:360–368. 10.1111/j.1349-7006.2011.02145.x 22066698

[20] *Vital Statistics Japan* *2018*. Health and Welfare Statistics Association, [Apr. 21, 2020 accessed]; Available from: http://www.e-stat.go.jp/SG1/estat/eStatTopPortal.do [in Japanese].

[21] *Population files for population-based cancer registry*. Center for Cancer Control and Information Services, National Cancer Center, [Apr. 21, 2020 accessed]; Available from: https://ganjoho.jp/reg_stat/statistics/dl/statistics_p05.html [in Japanese].

[22] *Estimated population (e-Stat)*. Bureau of Statistics, Ministry of Internal Affairs and Communications accessed]; Available from: https://www.e-stat.go.jp/stat-search/files?page=1&layout=datalist&toukei=00200524&tstat=000000090001&cycle=7&month=0&tclass1=000001011679&cycle_facet=tclass1%3Acycle [in Japanese].

[23] *Cancer Statistics in Japan; Table download*. Cancer Information Services, National Cancer Center, Japan, [Aug. 10, 2020 accessed]; Available from: https://ganjoho.jp/en/professional/statistics/table_download.html.

[24] Kim HJ, Fay MP, Feuer EJ, Midthune DN. Permutation tests for joinpoint regression with applications to cancer rates. Stat Med. 2000;19:335–351. 10.1002/(SICI)1097-0258(20000215)19:3<335::AID-SIM336>3.0.CO;2-Z 10649300

[25] *Graph Database*. Center for Cancer Control and Information Services, National Cancer Center, Japan, [May 18, 2020 accessed]; Available from: http://gdb.ganjoho.jp/graph_db/index?changeLang=Submit.

[26] Saito E, Hori M, Matsuda T, Yoneoka D, Ito Y, Katanoda K. Long-term trends in prostate cancer incidence by stage at diagnosis in Japan using the multiple imputation approach, 1993–2014. Cancer Epidemiol Biomarkers Prev. 2020;29:1222–1228. 10.1158/1055-9965.EPI-19-1228 32169995

[27] *Final report on the prostate cancer screening study (Heisei 13–17)*. Tokyo: The Japan Foundation for Prostate Research, 2011 [in Japanese].

[28] *Situation of the implementation of prostate cancer screening by municipality -June 2015 survey*. Tokyo: The Japan Foundation for Prostate Research, 2015 [in Japanese].

[29] Hosokawa M, Imazeki S, Mizunuma H, Kubota T, Hayashi K. Secular trends in age at menarche and time to establish regular menstrual cycling in Japanese women born between 1930 and 1985. BMC Womens Health. 2012;12:19. 10.1186/1472-6874-12-19 22800445 PMC3434095

[30] *Annual trends in number of birth, birth rate, male-to-female birth ratio, and total fertility rate*. e-Stat (Portal site for Japanese Government Statistics), [Nov. 13, 2020 accessed]; Available from: https://www.e-stat.go.jp/en/dbview?sid=0003214664 [in Japanese].

[31] Toyoda Y, Tabuchi T, Nakayama T, . Trends in the clinical stage distribution of breast cancer in Osaka, Japan. Breast Cancer. 2018;25:250–256. 10.1007/s12282-017-0807-7 29027114

[32] Pham TM, Quy PN, Horimatsu T, . Premature mortality due to stomach cancer in Japan: a nationwide analysis from 1980 to 2015. Ann Epidemiol. 2020;47:19–24. 10.1016/j.annepidem.2020.05.012 32713503

[33] Wang C, Nishiyama T, Kikuchi S, . Changing trends in the prevalence of H. pylori infection in Japan (1908–2003): a systematic review and meta-regression analysis of 170,752 individuals. Sci Rep. 2017;7:15491. 10.1038/s41598-017-15490-7 29138514 PMC5686167

[34] Koizumi S, Motoyama S, Watanabe N, Matsuhashi T, Iijima K. Chronological changes in the gastric cancer subsite in Akita, Japan: the trends from the data of a hospital-based registration system. Tohoku J Exp Med. 2018;246:131–140. 10.1620/tjem.246.131 30369514

[35] Tsuda M, Asaka M, Kato M, . Effect on Helicobacter pylori eradication therapy against gastric cancer in Japan. Helicobacter. 2017;22:e12415. 10.1111/hel.12415 28771894 PMC5655764

[36] Lagergren J. Adenocarcinoma of oesophagus: what exactly is the size of the problem and who is at risk? Gut. 2005;54(Suppl 1):i1–i5. 10.1136/gut.2004.041517 15711002 PMC1867797

[37] Tanaka H, Uera F, Tsukuma H, Ioka A, Oshima A. Distinctive change in male liver cancer incidence rate between the 1970s and 1990s in Japan: comparison with Japanese-Americans and US whites. Jpn J Clin Oncol. 2007;37:193–196. 10.1093/jjco/hyl148 17332055

[38] Lange CM, Jacobson IM, Rice CM, Zeuzem S. Emerging therapies for the treatment of hepatitis C. EMBO Mol Med. 2014;6:4–15. 10.1002/emmm.201303131 24106239 PMC3936496

[39] Cortez KJ, Kottilil S. Beyond interferon: rationale and prospects for newer treatment paradigms for chronic hepatitis C. Ther Adv Chronic Dis. 2015;6:4–14. 10.1177/2040622314551934 25553238 PMC4269609

[40] Suzuki T, Nakashima K, Chida T, Ito M. Advances in drug development for hepatitis C. Uirusu. 2015;65:239–244 [in Japanese]. 10.2222/jsv.65.239 27760922

[41] *Diet, Nutrition, Physical Activity and Cancer: a Global Perspective. Continuous Update Project Expert Report*. World Cancer Research Fund/American Institute for Cancer Research, [Nov. 13, 2020 accessed]; Available from: https://www.wcrf.org/dietandcancer.

[42] Rawla P, Sunkara T, Thandra KC, Barsouk A. Epidemiology of gallbladder cancer. Clin Exp Hepatol. 2019;5:93–102. 10.5114/ceh.2019.85166 31501784 PMC6728871

[43] Sharma A, Sharma KL, Gupta A, Yadav A, Kumar A. Gallbladder cancer epidemiology, pathogenesis and molecular genetics: recent update. World J Gastroenterol. 2017;23:3978–3998. 10.3748/wjg.v23.i22.3978 28652652 PMC5473118

[44] Kumar S, Kumar S, Kumar S. Infection as a risk factor for gallbladder cancer. J Surg Oncol. 2006;93:633–639. 10.1002/jso.20530 16724347

[45] Khan SA, Tavolari S, Brandi G. Cholangiocarcinoma: epidemiology and risk factors. Liver Int. 2019;39(Suppl 1):19–31. 10.1111/liv.14095 30851228

[46] Kinoshita FL, Ito Y, Morishima T, Miyashiro I, Nakayama T. Sex differences in lung cancer survival: long-term trends using population-based cancer registry data in Osaka, Japan. Jpn J Clin Oncol. 2017;47:863–869. 10.1093/jjco/hyx094 28903532

[47] Toyoda Y, Nakayama T, Ioka A, Tsukuma H. Trends in lung cancer incidence by histological type in Osaka, Japan. Jpn J Clin Oncol. 2008;38:534–539. 10.1093/jjco/hyn072 18689853 PMC2525496

[48] Ito H, Matsuo K, Tanaka H, . Nonfilter and filter cigarette consumption and the incidence of lung cancer by histological type in Japan and the United States: analysis of 30-year data from population-based cancer registries. Int J Cancer. 2011;128:1918–1928. 10.1002/ijc.25531 20589676

[49] *The Health Consequences of Smoking - 50 Years of Progress A Report of the Surgeon General*. U.S. Department of Health and Human Services, Centers for Disease Control and Prevention, Coordinating Center for Health Promotion, National Center for Chronic Disease Prevention and Health Promotion, Office on Smoking and Health, [Nov. 13, 2020 accessed]; Available from: https://www.cdc.gov/tobacco/data_statistics/sgr/50th-anniversary/index.htm.

[50] *Smoking and Health Report of the Committee on Health Effects of Smoking*. Ministry of Health, Labour and Welfare, [Jan. 8, 2019 accessed]; Available from: https://www.mhlw.go.jp/stf/shingi2/0000135586.html [in Japanese].

[51] Luostarinen T, Apter D, Dillner J, . Vaccination protects against invasive HPV-associated cancers. Int J Cancer. 2018;142:2186–2187. 10.1002/ijc.31231 29280138

[52] Smith M, Canfell K. Impact of the Australian National Cervical Screening Program in women of different ages. Med J Aust. 2016;205:359–364. 10.5694/mja16.00289 27736623

[53] Hanley SJ, Yoshioka E, Ito Y, Kishi R. HPV vaccination crisis in Japan. Lancet. 2015;385:2571. 10.1016/S0140-6736(15)61152-7 26122153

[54] Tsuda K, Yamamoto K, Leppold C, . Trends of media coverage on human papillomavirus vaccination in Japanese newspapers. Clin Infect Dis. 2016;63:1634–1638. 10.1093/cid/ciw647 27660235

[55] Simms KT, Hanley SJB, Smith MA, Keane A, Canfell K. Impact of HPV vaccine hesitancy on cervical cancer in Japan: a modelling study. Lancet Public Health. 2020;5:e223–e234. 10.1016/S2468-2667(20)30010-4 32057317

[56] Luo G, Zhang Y, Guo P, Ji H, Xiao Y, Li K. Global patterns and trends in pancreatic cancer incidence: age, period, and birth cohort analysis. Pancreas. 2019;48:199–208. 10.1097/MPA.0000000000001230 30589831

[57] Lucas AL, Malvezzi M, Carioli G, . Global trends in pancreatic cancer mortality from 1980 through 2013 and predictions for 2017. Clin Gastroenterol Hepatol. 2016;14(10):1452–1462.e4. 10.1016/j.cgh.2016.05.034 27266982 PMC5028258

[58] Welch HG, Black WC. Overdiagnosis in cancer. J Natl Cancer Inst. 2010;102:605–613. 10.1093/jnci/djq099 20413742

[59] Srivastava S, Koay EJ, Borowsky AD, . Cancer overdiagnosis: a biological challenge and clinical dilemma. Nat Rev Cancer. 2019;19:349–358. 10.1038/s41568-019-0142-8 31024081 PMC8819710

[60] Li R, Wang Y, Du L. A rapidly increasing trend of thyroid cancer incidence in selected East Asian countries: joinpoint regression and age-period-cohort analyses. Gland Surg. 2020;9:968–984. 10.21037/gs-20-97 32953606 PMC7475344

[61] La Vecchia C, Malvezzi M, Bosetti C, . Thyroid cancer mortality and incidence: a global overview. Int J Cancer. 2015;136:2187–2195. 10.1002/ijc.29251 25284703

[62] Ripping TM, Ten Haaf K, Verbeek ALM, van Ravesteyn NT, Broeders MJM. Quantifying Overdiagnosis in Cancer Screening: A Systematic Review to Evaluate the Methodology. J Natl Cancer Inst. 2017;109. 10.1093/jnci/djx060 29117353

[63] Damber JE. Decreasing mortality rates for prostate cancer: possible role of hormonal therapy? BJU Int. 2004;93:695–701. 10.1111/j.1464-410X.2003.04713.x 15049974

[64] Baade PD, Youlden DR, Cramb SM, Dunn J, Gardiner RA. Epidemiology of prostate cancer in the Asia-Pacific region. Prostate Int. 2013;1:47–58. 10.12954/PI.12014 24223402 PMC3814115

[65] Carioli G, Malvezzi M, Bertuccio P, . Cancer mortality in the elderly in 11 countries worldwide, 1970–2015. Ann Oncol. 2019;30:1344–1355. 10.1093/annonc/mdz178 31147682

[66] Chihara D, Ito H, Matsuda T, . Differences in incidence and trends of haematological malignancies in Japan and the United States. Br J Haematol. 2014;164:536–545. 10.1111/bjh.12659 24245986 PMC3907701

[67] Chihara D, Ito H, Matsuda T, . Decreasing trend in mortality of chronic myelogenous leukemia patients after introduction of imatinib in Japan and the U.S. Oncologist. 2012;17:1547–1550. 10.1634/theoncologist.2012-0197 22971523 PMC3528387

[68] Chihara D, Ito H, Matsuda T, . Association between decreasing trend in the mortality of adult T-cell leukemia/lymphoma and allogeneic hematopoietic stem cell transplants in Japan: analysis of Japanese vital statistics and Japan Society for Hematopoietic Cell Transplantation (JSHCT). Blood Cancer J. 2013;3:e159. 10.1038/bcj.2013.57 24241399 PMC3880440

[69] Usui Y, Ito H, Koyanagi Y, . Changing trend in mortality rate of multiple myeloma after introduction of novel agents: a population-based study. Int J Cancer. 2020;147(11):3102–3109. 10.1002/ijc.33135 32506433

[70] Allemani C, Matsuda T, Di Carlo V, ; CONCORD Working Group. Global surveillance of trends in cancer survival 2000–14 (CONCORD-3): analysis of individual records for 37 513 025 patients diagnosed with one of 18 cancers from 322 population-based registries in 71 countries. Lancet. 2018;391(10125):1023–1075. 10.1016/S0140-6736(17)33326-3 29395269 PMC5879496

[71] Yoshimura A, Ito H, Nishino Y, . Recent Improvement in the Long-term Survival of Breast Cancer Patients by Age and Stage in Japan. J Epidemiol. 2018;28:420–427. 10.2188/jea.JE20170103 29479003 PMC6143379

[72] Oze I, Ito H, Nishino Y, . Trends in Small-Cell Lung Cancer Survival in 1993–2006 Based on Population-Based Cancer Registry Data in Japan. J Epidemiol. 2019;29:347–353. 10.2188/jea.JE20180112 30449770 PMC6680055

[73] Inoue S, Hosono S, Ito H, ; J-CANSIS Research Group. Improvement in 5-Year Relative Survival in Cancer of the Corpus Uteri From 1993–2000 to 2001–2006 in Japan. J Epidemiol. 2018;28(2):75–80. 10.2188/jea.JE20170008 29109365 PMC5792230

[74] Nakagawa H, Ito H, Hosono S, . Changes in trends in colorectal cancer incidence rate by anatomic site between 1978 and 2004 in Japan. Eur J Cancer Prev. 2017;26:269–276. 10.1097/CEJ.0000000000000255 27149637

[75] Wong MCS, Huang J, Lok V, . Differences in incidence and mortality trends of colorectal cancer worldwide based on sex, age, and anatomic location. Clin Gastroenterol Hepatol. 2020. 10.1016/j.cgh.2020.02.026 32088300

[76] Takayama K. Progress of medical therapy for advanced lung cancer. J Kyoto Prefectural Univ Med. 2016;125:19–25 [in Japanese].

[77] Shigeta S, Shida M, Nagase S, . Epidemiological guideline influence on the therapeutic trend and patient outcome of uterine cervical cancer in Japan: Japan society of gynecologic oncology guideline evaluation committee project. Gynecol Oncol. 2020;159:248–255. 10.1016/j.ygyno.2020.07.023 32718728

[78] Utada M, Chernyavskiy P, Lee WJ, . Increasing risk of uterine cervical cancer among young Japanese women: comparison of incidence trends in Japan, South Korea and Japanese-Americans between 1985 and 2012. Int J Cancer. 2019;144:2144–2152. 10.1002/ijc.32014 30474210 PMC7478999

[79] Yagi A, Ueda Y, Kakuda M, . Epidemiologic and clinical analysis of cervical cancer using data from the population-based Osaka Cancer Registry. Cancer Res. 2019;79:1252–1259. 10.1158/0008-5472.CAN-18-3109 30635276

[80] Shigeta S, Nagase S, Mikami M, . Assessing the effect of guideline introduction on clinical practice and outcome in patients with endometrial cancer in Japan: a project of the Japan Society of Gynecologic Oncology (JSGO) guideline evaluation committee. J Gynecol Oncol. 2017;28:e76. 10.3802/jgo.2017.28.e76 29027394 PMC5641526

[81] Kato H, Nakajima M. Treatments for esophageal cancer: a review. Gen Thorac Cardiovasc Surg. 2013;61:330–335. 10.1007/s11748-013-0246-0 23568356

[82] Kato K, Chiba T, Shimada T, Shibuya D. Management of screening endoscopy for population-based screening for gastric cancer. Gastroenterological Endoscopy. 2016;58:2251–2261.

[83] Sugiyama T. Gynecologic cancers. Jpn J Chemotherapy. 2006;54:239–248 [in Japanese].

[84] Kigawa J, Katabuchi H, Yaegashi N. Evaluation and revision of treatment guidelines for ovarian cancer (2010). Jpn J Chemotherapy. 2010;37:617–619 [in Japanese]. 20414017

[85] Aoe J, Ito Y, Fukui K, . Long-term trends in sex difference in bladder cancer survival 1975–2009: A population-based study in Osaka, Japan. Cancer Med. 2020;9(19):7330–7340. 10.1002/cam4.3382 32794368 PMC7541165

[86] Antoni S, Ferlay J, Soerjomataram I, Znaor A, Jemal A, Bray F. Bladder cancer incidence and mortality: a global overview and recent trends. Eur Urol. 2017;71:96–108. 10.1016/j.eururo.2016.06.010 27370177

[87] Atkins MB, Choueiri TK. *Epidemiology, pathology, and pathogenesis of renal cell carcinoma*. [No. 16, 2020 accessed]; Available from: https://www.uptodate.com/contents/epidemiology-pathology-and-pathogenesis-of-renal-cell-carcinoma.

[88] Bagheri MH, Ahlman MA, Lindenberg L, . Advances in medical imaging for the diagnosis and management of common genitourinary cancers. Urol Oncol. 2017;35:473–491. 10.1016/j.urolonc.2017.04.014 28506596 PMC5931389

[89] Pal SK, Ghate SR, Li N, . Real-world survival outcomes and prognostic factors among patients receiving first targeted therapy for advanced renal cell carcinoma: a SEER-Medicare Database Analysis. Clin Genitourin Cancer. 2017;15:e573–e582. 10.1016/j.clgc.2016.12.005 28139444

[90] Washio M, Mori M, Khan M, ; JACC Study Group. Diabetes mellitus and kidney cancer risk: the results of Japan Collaborative Cohort Study for Evaluation of Cancer Risk (JACC Study). Int J Urol. 2007;14(5):393–397. 10.1111/j.1442-2042.2007.01744.x 17511719

[91] Washio M, Mori M, Sakauchi F, ; JACC Study Group. Risk factors for kidney cancer in a Japanese population: findings from the JACC Study. J Epidemiol. 2005;15(Suppl 2):S203–S211. 10.2188/jea.15.S203 16127235 PMC8639037

