# Supplementary material for: Updated Trends in Cancer in Japan: Incidence in 1985–2015 and Mortality in 1958–2018—A Sign of Decrease in Cancer Incidence
Source: J Epidemiol. 2021 Jul 5;31(7):426–50. doi: 10.2188/jea.JE20200416 (PMC8187612; doi:10.2188/jea.JE20200416)
Supplement: Supplementary file 1 [file je-31-426-s001.pdf]

eTable 1. Results of joinpoint regression analysis on the trends in all-cancer incidence: data from three prefectures (1985–2015): Under 75 years old[a]

Sex	Cancer site	ICD-10	Number of joinpoints	Line segment		Annual % change	95% confidence interval		
				Start	End		Lower	Upper	
Male and female	All cancers	C00-C96	2	1985	2005	0.7	0.5	0.8	*
				2005	2010	2.3	0.5	4.1	*
				2010	2015	-0.5	-1.7	0.7	
	All cancers excluding stomach	C00-C96 (excluding C16)	3	1985	1996	2.1	1.6	2.5	*
				1996	2000	0.0	-2.7	2.7	
				2000	2010	2.3	1.8	2.8	*
				2010	2015	0.1	-1.0	1.2	
	All cancers excluding stomach and liver	C00-C96 (excluding C16 and C22)	2	1985	2005	1.7	1.5	1.9	*
				2005	2010	3.2	1.4	5.1	*
				2010	2015	0.2	-1.1	1.4	
Male	All cancers	C00-C96	3	1985	1996	1.0	0.6	1.5	*
				1996	2000	-1.6	-4.6	1.6	
				2000	2010	1.3	0.7	1.8	*
				2010	2015	-1.2	-2.5	0.2	
	All cancers excluding stomach	C00-C96 (excluding C16)	3	1985	1996	2.1	1.6	2.6	*
				1996	2000	-1.0	-4.1	2.3	
				2000	2010	1.9	1.3	2.5	*
				2010	2015	-0.9	-2.2	0.5	
	All cancers excluding stomach and liver	C00-C96 (excluding C16 and C22)	3	1985	1996	2.3	1.7	2.8	*
				1996	2000	-0.5	-3.9	3.1	
				2000	2010	2.4	1.8	3.0	*
				2010	2015	-0.5	-1.9	1.0	
	All cancers excluding prostate	C00-C96 (excluding C61)	3	1985	1995	1.0	0.5	1.4	*
				1995	2005	-1.0	-1.6	-0.5	*
				2005	2009	1.9	-0.9	4.9	
				2009	2015	-1.2	-2.2	-0.3	*
Female	All cancers	C00-C96	2	1985	2004	0.9	0.7	1.1	*
				2004	2010	2.7	1.5	4.0	*
				2010	2015	0.5	-0.7	1.7	
	All cancers excluding stomach	C00-C96 (excluding C16)	2	1985	2005	1.7	1.6	1.9	*
				2005	2010	3.4	1.4	5.4	*
				2010	2015	0.5	-0.8	1.9	
	All cancers excluding stomach and liver	C00-C96 (excluding C16 and C22)	2	1985	2005	1.8	1.6	2.0	*
				2005	2010	3.7	1.8	5.6	*
				2010	2015	0.7	-0.6	2.0	
	All cancers excluding breast	C00-C96 (excluding C50)	1	1985	2004	0.3	0.1	0.5	*
				2004	2015	1.3	0.9	1.7	*

ICD-10, International Classification of Disease, version 10.

[a] Yamagata, Fukui, and Nagasaki Prefectures.

* Annual % change was statistically significantly different from zero (P<0.05).

eTable 2A. Results of joinpoint regression analysis on the trends in site-specific cancer incidence: data from three prefectures (1985–2015); Male, including cis[a]

Cancer site	ICD-10	Number of joinpoints	Line segment		Annual % change	95% confidence interval	
			Start	End		Lower	Upper
All cancers (including cis)	C00-C96 D00-D09	3	1985	1996	1.4	1.0	1.9 *
		3	1996	2000	-1.0	-3.7	1.8
		3	2000	2010	1.9	1.4	2.3 *
		3	2010	2015	-0.8	-1.9	0.3
Esophagus (including cis)	C15 D001	0	1985	2015	1.5	1.2	1.7 *
Colon (including cis)	C18 D010	2	1985	1996	9.1	7.6	10.7 *
			1996	2000	-2.6	-9.9	5.2
			2000	2015	2.2	1.6	2.8 *
Rectum (including cis)	C19-C20 D011-D012	1	1985	1993	5.5	2.8	8.3 *
			1993	2015	1.3	0.8	1.7 *
Colon/rectum (including cis)	C18-C20 D010-D012	2	1985	1995	7.1	5.8	8.4 *
			1995	2004	0.0	-1.3	1.2
			2004	2015	2.4	1.7	3.2 *
Lung, trachea (including cis)	C33-C34 D021-D022	1	1985	2010	0.8	0.6	0.9 *
			2010	2015	-2.1	-3.7	-0.4 *
Skin (including cis)	C43-C44 D030-D049	0	1985	2015	2.9	2.4	3.3 *
Breast (including cis)	C50 D05	0	1985	2015	2.1	0.7	3.5 *
Bladder (including cis)	C67 D090	0	1985	2015	2.1	1.8	2.4 *

cis, carcinoma in situ; ICD-10, International Classification of Disease, version 10.

[a] Yamagata, Fukui, and Nagasaki Prefectures.

* Annual % change was statistically significantly different from zero ($P < 0.05$).

eTable 2B. Results of joinpoint regression analysis on the trends in site-specific cancer incidence: data from three prefectures (1985–2015); Female, including CIS

Cancer site	ICD-10	Number of joinpoints	Line segment		Annual % change	95% confidence interval		
			Start	End		Lower	Upper	
All cancers (including cis)	C00-C96 D00-D09	2	1985	2004	1.2	1.0	1.3	*
		2	2004	2012	3.7	3.0	4.4	*
		2	2012	2015	0.7	-1.8	3.2	
Esophagus (including cis)	C15 D001	1	1985	1996	-2.9	-5.4	-0.4	*
			1996	2015	3.0	1.9	4.1	*
Colon (including cis)	C18 D010	1	1985	1995	5.0	3.7	6.3	*
			1995	2015	1.7	1.4	2.0	*
Rectum (including cis)	C19-C20 D011-D012	2	1985	1998	2.6	2.1	3.1	*
			1998	2004	-2.0	-3.8	-0.2	*
			2004	2015	2.7	2.1	3.2	*
Colon/rectum (including cis)	C18-C20 D010-D012	2	1985	1995	4.3	3.5	5.1	*
			1995	2004	0.3	-0.5	1.2	
			2004	2015	2.3	1.8	2.8	*
Lung, trachea (including cis)	C33-C34 D021-D022	0	1985	2015	1.9	1.7	2.1	*
Skin (including cis)	C43-C44 D030-D049	2	1985	1997	-0.3	-1.8	1.2	
			1997	2004	6.9	3.2	10.7	*
			2004	2015	1.9	0.6	3.2	*
Breast (including cis)	C50 D05	2	1985	2002	3.9	3.5	4.3	*
			2002	2010	5.9	4.7	7.0	*
			2010	2015	1.2	-0.4	2.9	
Uterus (including cis)	C53-C55 D06	3	1985	1988	-6.7	-16.3	3.9	
			1988	2006	3.0	2.2	3.8	*
			2006	2012	12.2	7.9	16.6	*
			2012	2015	2.7	-4.9	10.9	
Cervix uteri (including cis)	C53 D06	4	1985	1998	0.2	-1.0	1.4	
			1998	2002	8.7	-2.4	21.0	
			2002	2006	-0.1	-9.8	10.7	
			2006	2012	14.3	9.9	18.9	*
			2012	2015	2.7	-4.6	10.6	
Bladder (including cis)	C67 D090	0	1985	2015	1.6	1.2	2.1	*

cis, carcinoma in situ; ICD-10, International Classification of Disease, version 10.

[a] Yamagata, Fukui, and Nagasaki Prefectures.

* Annual % change was statistically significantly different from zero (P<0.05).

eTable 3. Results of joinpoint regression analysis on the trends in all-cancer mortality: national data (1958–2018): Under 75 years old

Sex	Cancer site	ICD-10	Number of joinpoints	Line segment		Annual % change	95% confidence interval		
				Start	End		Lower	Upper	
Male and female	All cancers	C00-C97	4	1958	1967	-0.1	-0.3	0.2	
				1967	1976	-1.0	-1.3	-0.8	*
				1976	1993	-0.7	-0.7	-0.6	*
				1993	1997	0.5	-0.4	1.5	
				1997	2018	-1.8	-1.9	-1.8	*
	All cancers excluding stomach	C00-C97 (excluding C16)	1	1958	1997	0.6	0.6	0.7	*
				1997	2018	-1.5	-1.5	-1.4	*
	All cancers excluding stomach and liver	C00-C97 (excluding C16 and C22)	5	1958	1964	1.6	1.0	2.2	*
				1964	1980	0.7	0.5	0.8	*
				1980	1992	0.2	0.0	0.3	*
				1992	1996	1.2	0.2	2.2	*
				1996	2016	-0.8	-0.9	-0.8	*
				2016	2018	-1.9	-3.8	0.1	
Male	All cancers	C00-C97	4	1958	1967	0.4	0.1	0.7	*
				1967	1975	-0.5	-0.9	-0.1	*
				1975	1997	-0.1	-0.2	0.0	*
				1997	2016	-2.2	-2.3	-2.1	*
				2016	2018	-3.5	-5.8	-1.1	*
	All cancers excluding stomach	C00-C97 (excluding C16)	2	1958	1985	1.7	1.6	1.7	*
				1985	1997	0.8	0.6	1.0	*
				1997	2018	-1.9	-2.0	-1.9	*
	All cancers excluding stomach and liver	C00-C97 (excluding C16 and C22)	5	1958	1964	3.2	2.5	4.0	*
				1964	1980	1.6	1.5	1.8	*
				1980	1997	0.7	0.7	0.8	*
				1997	2009	-1.3	-1.4	-1.1	*
				2009	2016	-0.9	-1.2	-0.6	*
				2016	2018	-3.2	-5.2	-1.2	*
	All cancers excluding prostate	C00-C97 (excluding C61)	3	1958	1967	0.4	0.0	0.7	*
				1967	1975	-0.6	-1.0	-0.1	*
				1975	1997	-0.1	-0.2	-0.1	*
				1997	2018	-2.3	-2.3	-2.2	*
Female	All cancers	C00-C97	4	1958	1968	-0.6	-0.8	-0.4	*
				1968	1990	-1.5	-1.6	-1.5	*
				1990	1999	-0.4	-0.6	-0.2	*
				1999	2003	-1.9	-2.9	-0.9	*
				2003	2018	-1.1	-1.2	-1.0	*
	All cancers excluding stomach	C00-C97 (excluding C16)	2	1958	1992	-0.4	-0.4	-0.4	*
				1992	1996	0.9	-0.2	2.0	
				1996	2018	-0.8	-0.9	-0.8	*
	All cancers excluding stomach and liver	C00-C97 (excluding C16 and C22)	4	1958	1980	-0.2	-0.2	-0.1	*
				1980	1990	-0.6	-0.8	-0.4	*
				1990	1998	0.2	-0.1	0.5	
				1998	2003	-1.0	-1.6	-0.3	*
				2003	2018	-0.3	-0.4	-0.3	*
	All cancers excluding breast	C00-C97 (excluding C50)	5	1958	1968	-0.7	-0.9	-0.5	*
				1968	1992	-1.8	-1.8	-1.7	*
				1992	1997	-0.4	-1.0	0.2	
				1997	2009	-1.8	-2.0	-1.7	*
				2009	2014	-0.8	-1.4	-0.1	*
				2014	2018	-1.9	-2.6	-1.3	*

ICD-10, International Classification of Disease, version 10.

* Annual % change was statistically significantly different from zero ($P < 0.05$).

eFigure 1. Contribution of cancer sites to the changes in incidence; Under 75 years old

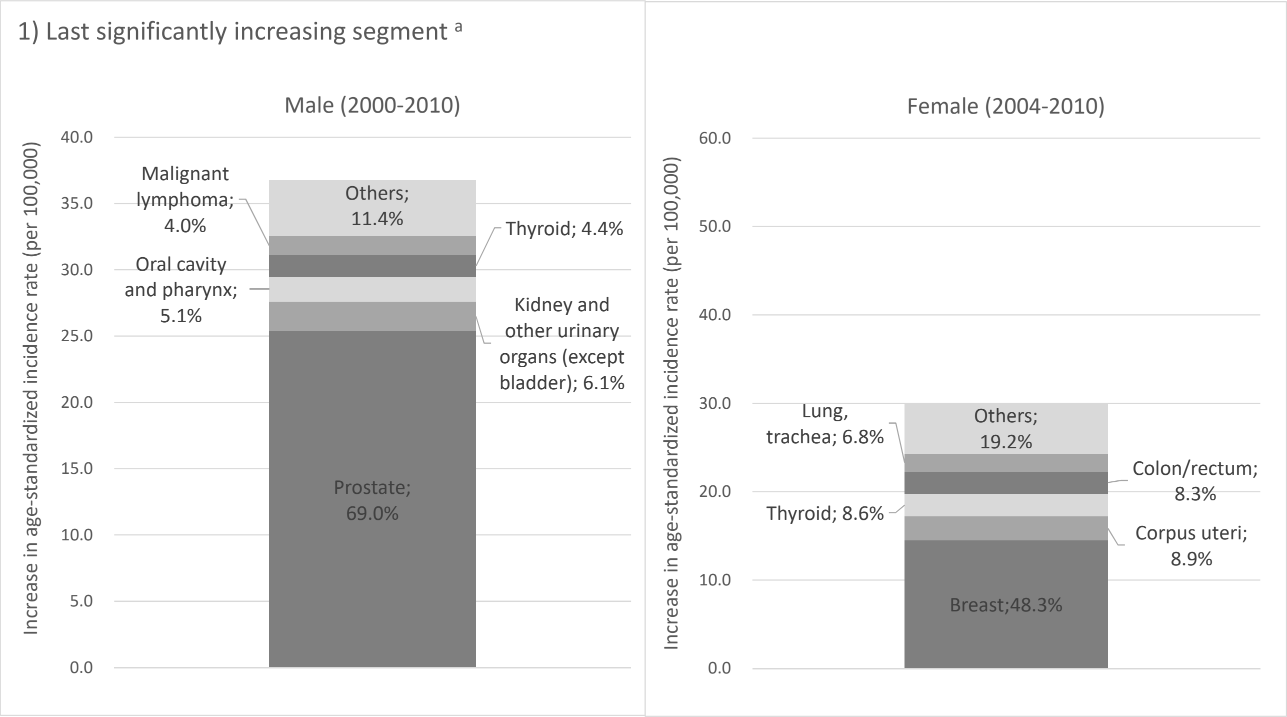

a. There was no significant change after 2010 both for males and females.

eFigure 2. Contribution of cancer sites to the decrease in mortality in recent 10 years (2009-2018); Under 75 years old

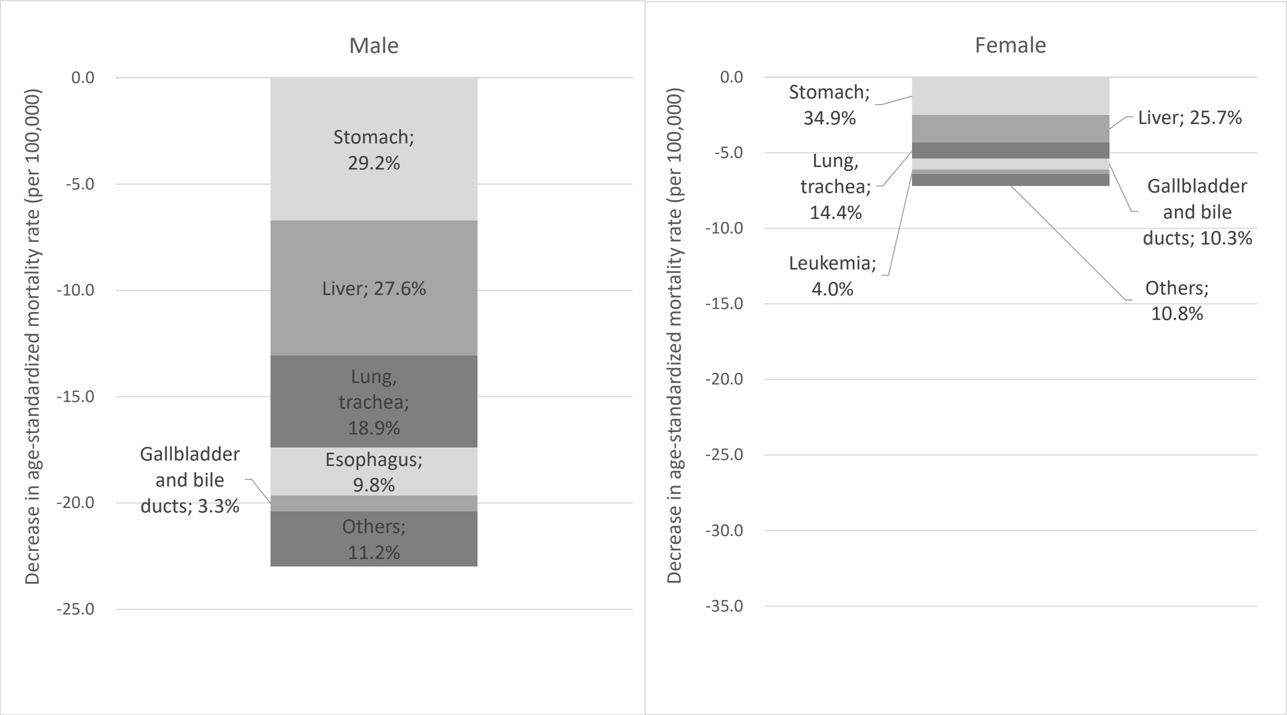

eFigure 3. Observed and modelled trends in cancer incidence (1985-2015) and mortality (1958-2018) rates: Cancers other than major and sub-major sites.

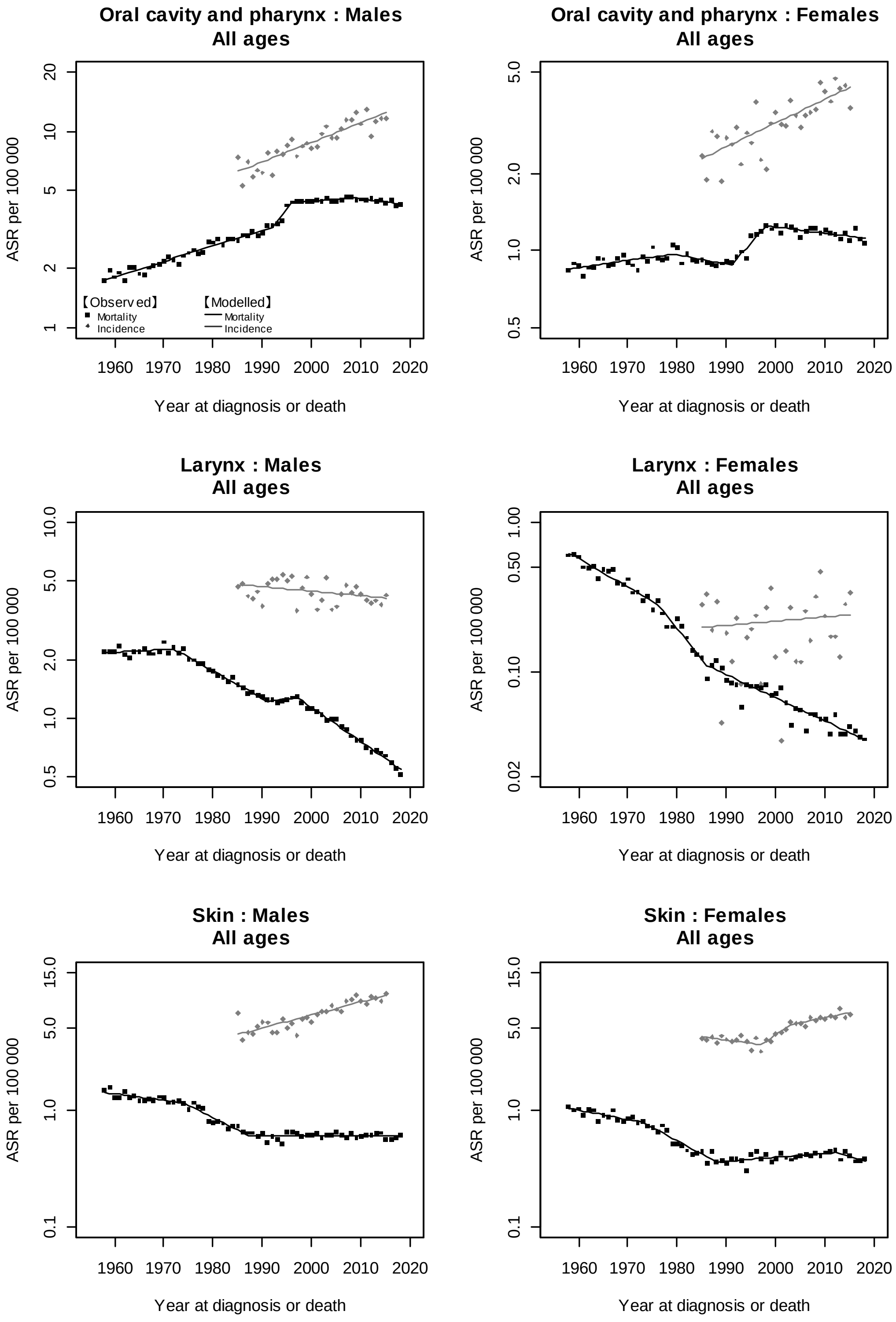

a. Incidence: data from Yamagata, Fukui, and Nagasaki Prefectures, Mortality: national data.

b. Standardized to Japanese model population in 1985.

eFigure 3. (Continued)

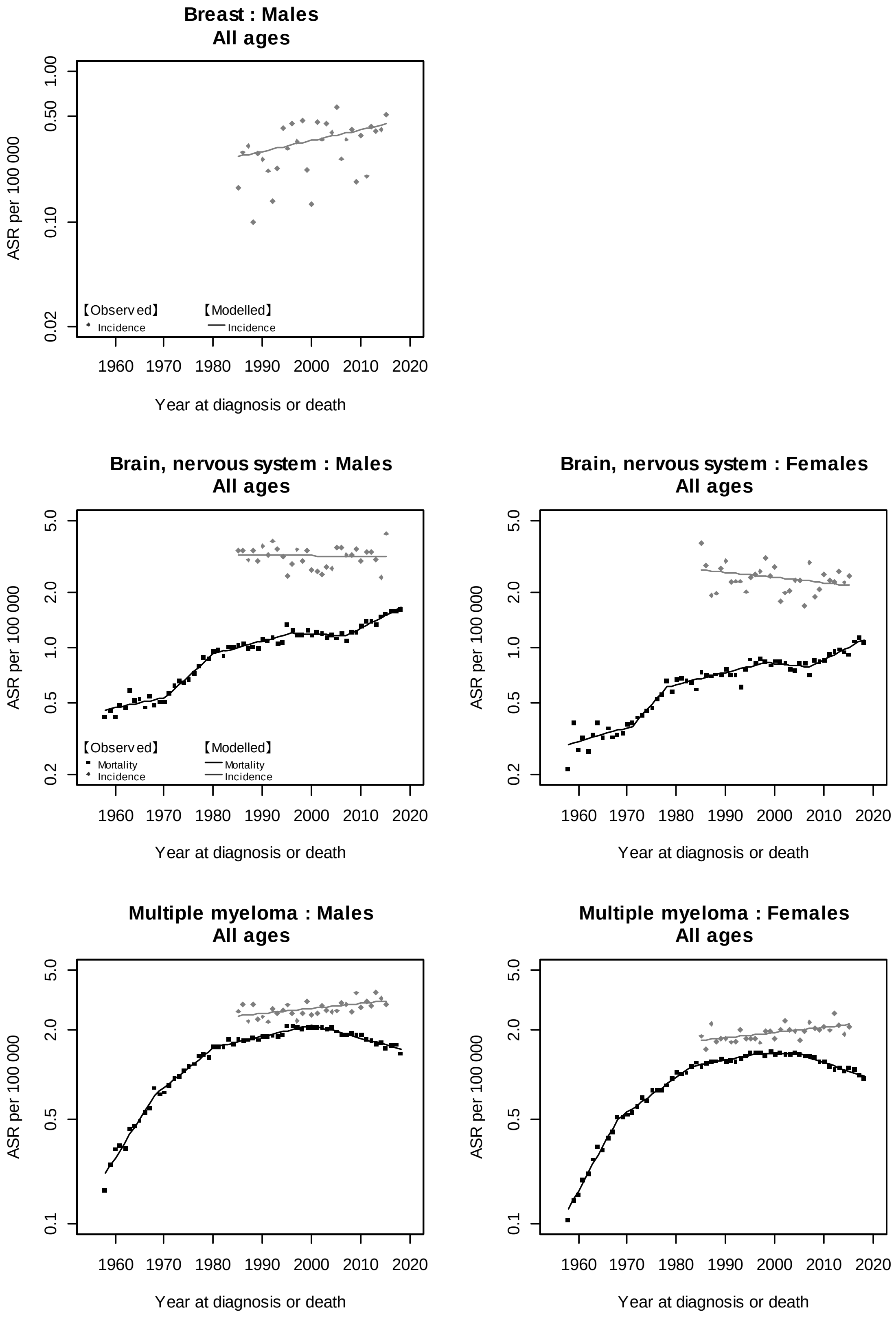

a. Incidence: data from Yamagata, Fukui, and Nagasaki Prefectures, Mortality: national data.

b. Standardized to Japanese model population in 1985.

eFigure 3. (Continued)

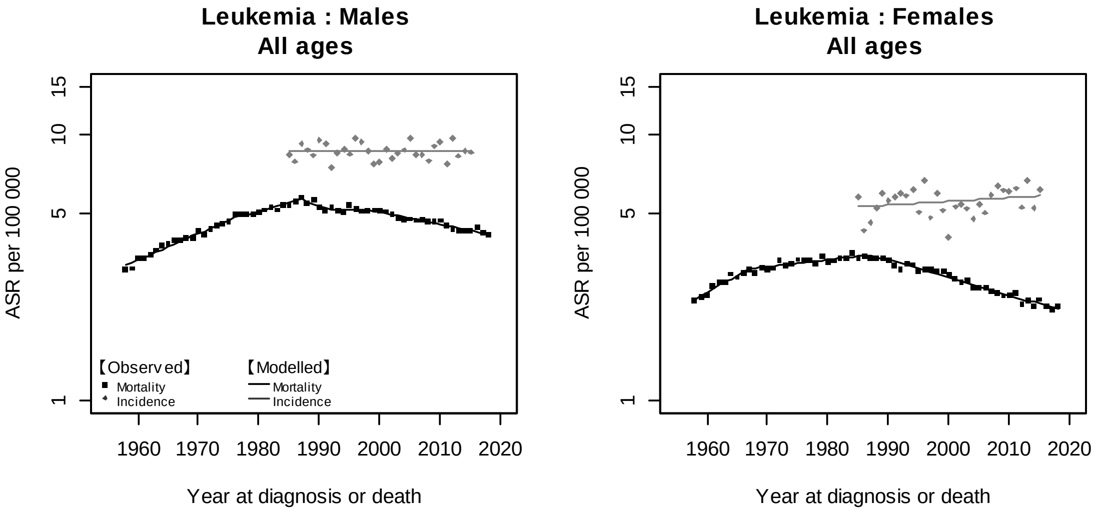

a. Incidence: data from Yamagata, Fukui, and Nagasaki Prefectures, Mortality: national data.

b. Standardized to Japanese model population in 1985.

eFigure 4. Annual trends in the quality indexes of cancer incidence data from three Prefectures (1985–2015)

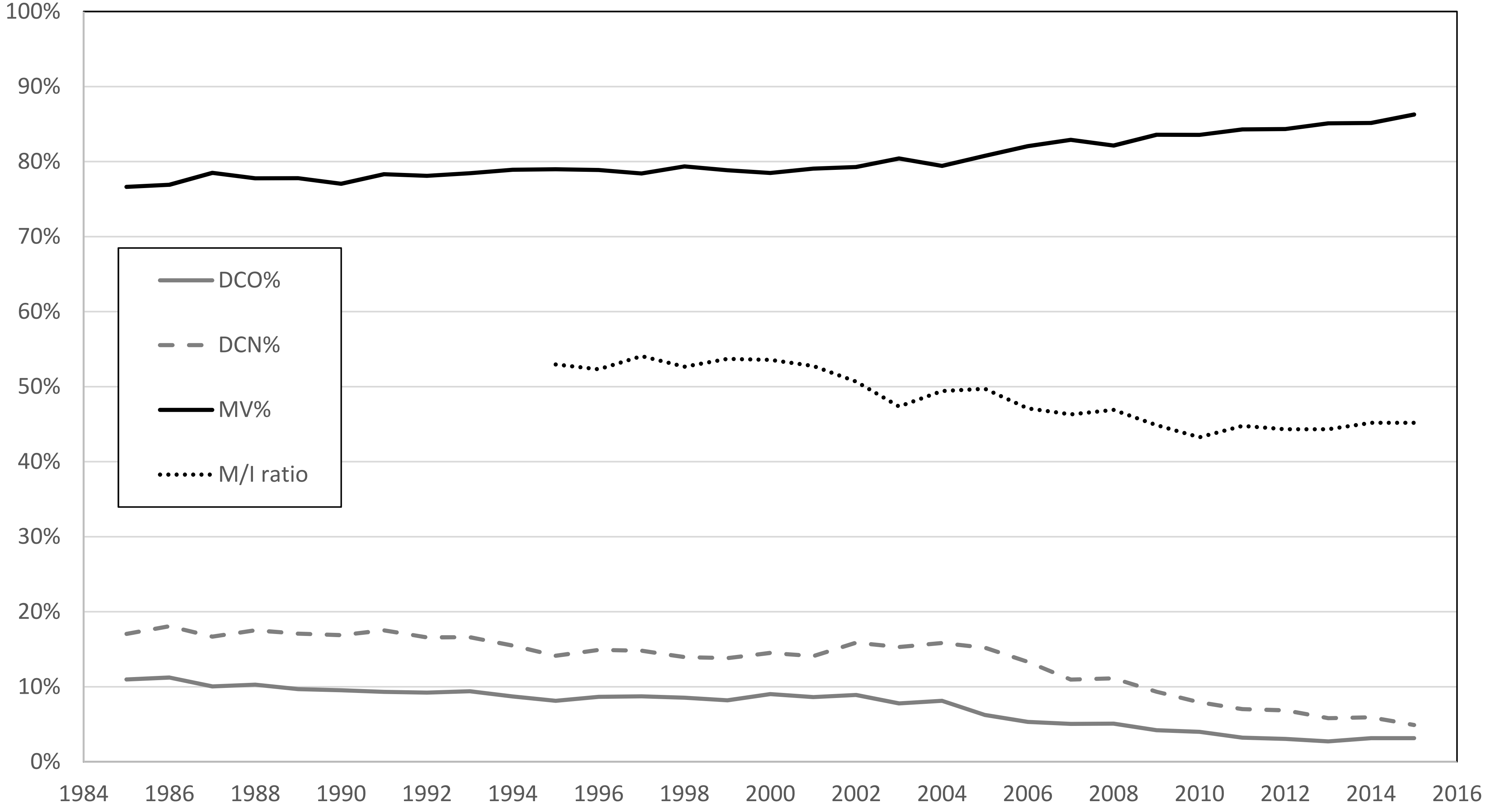

DCO: death certificate only; DCN: death certificate notification; MV: morphologically verified; M/I: mortality/incidence
